# Supplementary material for: The human microbiota is associated with cardiometabolic risk across the epidemiologic transition
Source: PLoS One. 2019 Jul 24;14(7):e0215262. doi: 10.1371/journal.pone.0215262 (PMC6656343; doi:10.1371/journal.pone.0215262)
Supplement: S1 File — (DOCX) [file pone.0215262.s001.docx]

**Supplementary results**

**Contributions of** **CM risk and environmental factors to the inter-individual dissimilarities in gut microbiota composition are diverse between countries**

Environmental factors, like gender, age, health cardiometabolic behavior, etc., may all contribute to shape the gut microbiota. We next investigated the contribution of each CM risk factor observed in the entire gut microbial dissimilarity matrix (weighted and unweighted UniFrac distance), compared to other participant and life style factors, including gender, age, BMI, sleep, smoking and alcohol consumption (**S1 Table**). For the weighted UniFrac distance, in the US, HDL concentration (R^2^=0.013, p=0.030), gender (R^2^=0.068, p=0.001), BMI (R^2^=0.031, p=0.002), smoking (R^2^=0.032, p=0.011), and alcohol consumption (R^2^=0.017, p=0.010) all contributed significantly to the gut microbiota composition and explained a greater proportion of the variance than HDL concentration. Among South Africans, only 3 CM risk factors, including waist circumference (R^2^=0.012, p=0.049), Elevated blood pressure (R^2^=0.022, p=0.004) and elevated fasting plasma glucose (R^2^=0.013, p=0.041), were significantly associated with gut microbial composition. In Ghana, BMI (R^2^=0.054, p=0.001, maximum), waist circumference (R^2^=0.026, p=0.001), smoking (R^2^=0.020, p=0.049), gender (R^2^=0.019, p=0.006), alcohol consumption (R^2^=0.013, p=0.039), Elevated blood pressure (R^2^=0.013, p=0.025, minimum), all contributed significantly to the gut microbial composition, respectively. For the unweighted UniFrac distance, environmental factors, gender (R^2^= 0.045, p=0.001), smoking (R^2^=0.031, p=0.010), and alcohol consumption (R^2^=0.018, p=0.001), all contributed significantly to the gut microbial composition in the US population. While among South Africans, CM risk factors including; waist circumference (R^2^=0.014, p=0.034) and Elevated blood pressure (R^2^=0.013, p=0.036), were significantly associated with gut microbial composition. Among Ghanaians for the unweighted UniFrac distance, BMI (R^2^=0.040, p=0.010), waist circumference (R^2^=0.019, p=0.001) and gender (R^2^=0.015, p=0.011), were significantly associated with the gut microbial composition. However, neither the CM risk factors, nor the environmental factors had significant effect on the gut microbial composition based on weighted or unweighted UniFrac distance. These results suggest that both CM risk and environmental factors contributed significantly to the gut microbial composition, and the influence pattern was different between countries and factors.

**Specific gut bacterial taxa** **are associated with individual CM risk factors**

We identified the fecal bacterial exact sequence variants (ESVs) that have significantly different abundance as a function of CM risk factors (**S6 and S7 Figs** and **S2** **Table).** For these differential ESVs with relative abundance higher than 1% in at least one group, across the entire cohort (adjusted for country, age, gender and BMI) or when stratified by gender (adjusted for country, BMI and age), we found that participants with a high waist circumference were significantly enriched with a bacterial ESV annotated to genus *Roseburia* (family Lachnospiraceae), which was also significantly enriched in female participants (p(fdr-corrected) <0.05) (**S6a Fig**). ESVs assigned to genus *Streptococcus* (family Lachnospiraceae)*, Coprococcus* (family Lachnospiraceae), *Blautia* (family Lachnospiraceae), and unclassified genus in family Lachnospiraceae were significantly enriched in male participants with a high waist circumference (p(fdr-corrected) <0.05) (**S6b Fig**). Conversely, 5 bacterial ESVs annotated to genus *Prevotella* (family Prevotellaceae), and family Enterobacteriaceae, Clostridiaceae, Peptostreptococcaceae were significantly enriched among participants with a normal waist circumference (p(fdr-corrected) <0.05) (**S6c Fig**). A single ESV annotated to family Enterobacteriaceae was significantly enriched among participants with a normal waist circumference in female and male participants, separately; while 1 bacterial ESV annotated to genus *Prevotella* (family Prevotellaceae) was only significantly enriched among participants with a normal waist circumference in male participants, not in female participants, and 2 bacterial ESVs annotated to family Clostridiaceae and 1 to genus *Oscillospira* (family Ruminococcaceae) were only significantly enriched among participants with a normal waist circumference in female participants, not in male participants (p(fdr-corrected) <0.05) (**S6b and 6c Figs**). One bacterial ESV annotated to family Enterobacteriaceae was significantly enriched in participants with elevated blood pressure, and also in just female participants (p(fdr-corrected) <0.05) (**S6d and 6e Figs**). Another 2 bacterial ESVs annotated to family Erysipelotrichaceae and Veillonellaceae, separately, were significantly enriched in people without elevated blood pressure only in female participants (p(fdr-corrected) <0.05) (**S6f Fig**). And 1 bacterial ESV annotated to family Enterobacteriaceae was significantly enriched in participants without elevated blood pressure, and also in just female participants (p(fdr-corrected) <0.05) (**S6f Fig**). Participants with elevated blood pressure had a significant enrichment in 1 bacterial ESV annotated to family Clostridiaceae only in female participants (p(fdr-corrected) <0.05) (**Fig S6f**). Besides, another 4 ESVs annotated to Parabacteroides (family Porphyromonadaceae), family Peptostreptococcaceae, genus *Oscillospira* (family Ruminococcaceae) and genus *Bacteroides* (family Bacteroidaceae), separately, were significantly enriched in female participants without elevated blood pressure (p(fdr-corrected) <0.05) (**S6f Fig**). Male participants without elevated blood pressure has one ESV annotated to genus *Faecalibacterium* (family Ruminococcaceae) significantly enriched (p(fdr-corrected) <0.05) (**S6g Fig**). 1 bacterial ESV annotated to genus *Prevotella* (family Prevotellaceae) was significantly enriched in participants without hypertriglyceridemia, and also just in female participants (p(fdr-corrected) <0.05) (**S6i and 6j Figs**). And female participants with hypertriglyceridemia were significantly enriched with 4 bacterial ESVs annotated to genus *Bacteroides* (family Bacteroidaceae)*,* *Parabacteroides* (family *Porphyromonadaceae)*, and genus *Coprococcus* (family Lachnospiraceae), separately (p(fdr-corrected) <0.05) (**S6j Fig**). Another 3 bacterial ESVs annotated to genus Oscillospira (family Ruminococcaceae), family Clostridiaceae, and genus *Catenibacterium* (family Erysipelotrichaceae), separately, were significantly enriched in only female participants without hypertriglyceridemia (p(fdr-corrected) <0.05) (**S6j Fig**). There was 1 bacterial ESV annotated to family Enterobacteriaceae significantly enriched in participants with low HDL concentration (p(fdr-corrected) <0.05) (**S6k Fig**).

**Specific gut bacterial taxa are differentially abundant** **between countries and individual** **CM risk factors.**

For these differential ESVs with relative abundance higher than 1% in at least one group, within each country (adjusted for gender, BMI and age), individuals with a high waist circumference were significantly enriched with a bacterial ESV annotated to genus *Dialister* (family Vellonellaceae). In Ghana, 1 bacterial ESV annotated to genus *Blautia* (family Lachnospiraceae), was significantly enriched in people with a low waist circumference. Jamaican participants with hyperglyceridemia had a significant enrichment in a bacterial ESV annotated to family Ruminococcaceae. 1 bacterial ESV annotated to genus *Oscillospira* (family Ruminococcaceae), was significantly enriched in people without hypertriglyceridemia in South Africa (p fdr-corrected) <0.05). No other statistically significant associations were observed (p(fdr-corrected) >0.05) (**S7 Fig** and **S2 Tables**).

**Gut bacterial function is associated with individual CM risk factors**

The 16S rRNA amplicon data was used to predict functional gene abundance in each dataset (Piphillin (*Shoko Iwai, Plos One, 2016,* *PMID:27820856*) to establish differences in the metabolic potential in several well-known pathways associated with CM, such as LPS biosynthesis, short-chain fatty acid (SCFA) metabolism, TMA N-oxide (TMAO) biosynthesis, and genes associated with secondary bile acid biosynthesis. No statistically significant differences in the genes associated with TMA N-oxide (TMAO) biosynthesis and secondary bile acid biosynthesis were observed between any CM risk factor in any country (p(fdr-corrected) >0.05).

*LPS biosynthesis pathways*. We identified the 39 of the 40 genes associated with the LPS biosynthesis from the KEGG (Kyoto Encyclopedia of Genes and Genomes) database in the predicted metagenomes. Among Ghanaians, the predicted abundances of 14 KOs were significantly enriched in participants with elevated blood pressure (p(fdr-corrected) <0.05). Also, O-antigen ligase (K02847) was significantly enriched in participants with a high waist circumference (p(fdr-corrected) <0.05), and D-glycero-alpha-D-manno-heptose-7-phosphate kinase (K07031) was significantly enriched in participants with a low waist circumference (p(fdr-corrected) <0.01). In the US, heptose L-phosphotransferase (K02848) and UDP-glucose/galactose:(glucosyl) LPS alpha-1,2-glucosyl/galactosyltransfe rase (K03276) were significantly enriched in participants with elevated fasted blood glucose (p(fdr-corrected) <0.05), and 3-deoxy-D-manno-octulosonate 8-phosphate phosphatase (KDO 8-P phosphatase) (K03270) was significantly enriched in participants with a high waist circumference (p(fdr-corrected) <0.01). Additionally, UDP-glucose:(heptosyl) LPS alpha-1,3-glucosyltransferase (K02844), heptosyltransferase III (K02849), and KDO II ethanolaminephosphotransferase (K12975) were significantly enriched in people with high HDL concentration (p(fdr-corrected) <0.05), while K02847 was significantly enriched in people with low HDL concentration (p(fdr-corrected) <0.05). Among Jamaicans, K02847 was significantly enriched in individuals with elevated fasted blood glucose (p(fdr-corrected) <0.05) and K07031 was significantly enriched in participants without elevated fasted blood glucose (p(fdr-corrected) <0.01), while D-glycero-D-manno-heptose 1,7-bisphosphate phosphatase (K03273) was significantly enriched in participants without elevated blood pressure (p(fdr-corrected) <0.01). Among South Africans, only 3-deoxy-D-manno-octulosonic acid kinase (K11211) were significantly enriched, and only in participants with low HDL concentration (p(fdr-corrected) <0.05) (**S8 Fig**).

*SCFA biosynthesis pathways*. The KEGG pathways involve in the microbial SCFAs biosynthesis pathways were also estimated, including pyruvate metabolism (ko00620), propanoate metabolism (ko00640), and butanoate metabolism (ko00650). Genes involved in the pyruvate metabolism and propanoate metabolism pathways were significantly enriched in people with a normal waist circumference in Ghana (p(fdr-corrected) <0.05). Interestingly, genes involved in all of the three pathways were significantly enriched in participants with a normal waist circumference in South Africa (p(fdr-corrected) <0.05). However, genes involved in all of the three pathways were significantly enriched in participants with hypertension in South Africa (p(fdr-corrected) <0.05). No significant differences were determined between any CM risk factor in the US and Jamaican population (p(fdr-corrected) >0.05) (**S9 Fig**).

The microbial biosynthesis of butyrate is mediated by four different pathways in the human microbiome: acetyl-CoA pathway, lysine pathway, glutarate pathway, and 4-aminobytyrate/succinate pathway *(Marius Vital, mBio, PMID: 24757212*). Each pathway has a different beginning substrate and produces different types of associated metabolites, and the manner of butyrate synthesis has been previously associated with disease states (*Maude M David, doi: https://doi.org/10.1101/319236*). For the whole cohort (adjusted for country, age, BMI and gender), crotonase (K01715) and β-hydroxybutyryl-CoA dehydrogenase (K00074) in the acetyl-CoA pathway, β-lysine-5,6-aminomutase α subunit (K01844), β-lysine-5,6-aminomutase β subunit (K18011) in the lysine pathway, glutaconyl-CoA decarboxylase (α, β subunits) (K01615) in the glutarate pathway, and 4-hydroxybutyryl-CoA dehydratase (K14534) in the 4-aminobytyrate/succinate pathway were significantly enriched in participants with a high waist circumference. Crotonase (K01692, K01782, K01825) in the acetyl-CoA pathway was also significantly enriched in participants with a normal waist circumference (p(fdr-corrected) <0.05). Thiolase (K00626), crotonase (K01715), and β-hydroxybutyryl-CoA dehydrogenase (K00074) in the acetyl-CoA pathway, β-lysine-5,6-aminomutase α subunit (K01844), β-lysine-5,6-aminomutase β subunit (K18011), and 3-aminobutyryl-CoA ammonia lyase (K18014) in the lysine pathway, glutaconyl-CoA decarboxylase (α, β subunits) (K01615) in the glutarate pathway, and 4-hydroxybutyryl-CoA dehydratase (K14534) in the 4-aminobytyrate/succinate pathway were significantly enriched in participants with hypertriglyceridemia (p(fdr-corrected) <0.05). Crotonase (K01782, K01825) in the acetyl-CoA pathway was significantly enriched in people without hypertriglyceridemia (p(fdr-corrected) <0.05). 3-aminobutyryl-CoA ammonia lyase (K18014) in the lysine pathway was significantly enriched in participants with elevated blood pressure (p(fdr-corrected) <0.05). Crotonase (K01782, K01825) in the acetyl-CoA pathway was significantly enriched in people without elevated blood pressure (p(fdr-corrected) < 0.05). Additionally, lysine-2,3-aminomutase (K01843) in the glutarate pathway was also significantly enriched in people with high HDL concentration (p(fdr-corrected) <0.05). Thiolase (K00626) in the acetyl-CoA pathway was significantly enriched in participants with low HDL concentration (p(fdr-corrected) <0.05). No other significant differences were identified for elevated fasted blood glucose in the whole cohort (p(fdr-corrected) >0.05) (**S10 Fig**).

**Predicted genes involved in SCFA biosynthesis pathways** **in gut microbiota between countries and individual CM risk factors.**

We also detected the differential relative abundance of the genes involved in the four different pathways for butyrate synthesis against CM risk factors within each country independently. For the waist circumference, all KOs predicted in the glutarate pathway, including glutaconate CoA transferase α subunit (K01039), glutaconate CoA transferase β subunit (K01040) and glutaconyl-CoA decarboxylase (α, β subunits) (K01615), and 4-hydroxybutyrate dehydrogenase (K18120) in the 4-aminobytyrate/Succinate succinate pathway were significantly enriched in participants with a high waist circumference in South Africa were significantly enriched in participants with a high waist circumference in South Africa (p(fdr-corrected) <0.05). Among Ghanaians, glutaconyl-CoA decarboxylase (α, β subunits) (K01615) in the glutarate pathway was significantly enriched in participants with a high waist circumference (p(fdr-corrected) < 0.05). 3-aminobutyryl-CoA ammonia (K18014) in the lysine pathway in South Africa and crotonase (K01692) in the Acetyl-CoA pathway in Jamaica were all significantly enriched among people with a normal waist circumference (p(fdr-corrected) < 0.05). No significant differences were found for waist circumference among the US cohort (p(fdr-corrected) > 0.05). For fasting blood glucose level, only crotonase (K01692) in the Acetyl-CoA pathway was significantly enriched in participants with hyperglyceridemia in US; while 3,5-diaminohexanoate dehydrogenase (K18012) in the lysine pathway was significantly enriched in people without hyperglyceridemia in Jamaica (p(fdr-corrected) <0.05). No significant differences were found for hyperglyceridemia in either South Africa or Ghana cohorts (p(fdr-corrected) > 0.05). For the blood pressure, glutaconyl-CoA decarboxylase (α, β subunits) (K01615) in the glutarate pathway was significantly enriched in people without Elevated blood pressure in South Africa; while 3,5-diaminohexanoate dehydrogenase (K18012), and 3-keto-5-aminohexanoate cleavage enzymes (K18013) in the lysine pathway were enriched in participants with Elevated blood pressure in Ghana (p(fdr-corrected) < 0.05). No significant differences were found for Elevated blood pressure in the US and Jamaican cohorts (p(fdr-corrected) > 0.05). For the HDL concentration, all KOs predicted in the glutarate pathway, including glutaconate CoA transferase α subunit (K01039), glutaconate CoA transferase β subunit (K01040), and glutaconyl-CoA decarboxylase (α, β subunits) (K01615) were significantly enriched in participants with low HDL concentrations in the US and South Africa. Additionally, β-hydroxybutyryl-CoA dehydrogenase (K00074) and crotonase (K01715) in the Acetyl-CoA pathway in US individuals, and 4-hydroxybutyrate dehydrogenase (K18120) in the 4-aminobytyrate/ succinate pathway in South Africans were also significantly associated with low HDL concentrations. No significant differences were found for HDL concentration in the Ghanaians (p(fdr-corrected) > 0.05). Finally, for the triglyceride concentration, only thiolase (K00626) in the Acetyl-CoA pathway was significantly enriched in participants with hypertriglyceridemia (p(fdr-corrected) < 0.05). No significant differences were found for hypertriglyceridemia in the US and South Africa population (p(fdr-corrected) > 0.05) (**S11 Fig**).

**Predicted genes involved in the TMA(O) biosynthesis pathways in gut microbiota between countries and** **individual CM risk factors.**

Microbial TMA(O) biosynthesis potential was predicted for each country independently. We identified the key genes of the main TMA-synthesis pathways, encoding choline TMA-lyase (*CutC*) and its activator *CutD* and carnitine oxygenase/reductase (*CntA/B*, *YeaW/X*) (*Craciun S, Proc Natl Acad Sci U S A. 2012,* PMID:23151509*;* *Zhu Y, Proc Natl Acad Sci U S A. 2014,* PMID:24591617*;* *Koeth RA, Cell Metab. 2014,* *PMID:25440057*). We could not find any differences in *CutC/D* abundance ratio between any CM risk factor in any country (p(fdr-corrected) > 0.05). The enzyme complex termed *CntA/B*, which shows close sequence similarity to *YeaW/X* (*Koeth RA, Cell Metab. 2014,* *PMID:25440057*) were not detected in the predicted metagenome for any of samples in this study.

**Predicted genes involved in secondary bile acid biosynthesis in gut microbiota between countries and individual CM risk factors.**

We also predicted enzymes involved in secondary bile acids biosynthesis. Only 7-alpha-hydroxysteroid dehydrogenase (K00076), choloylglycine hydrolase (K01442), bile acid-coenzyme A ligase (K15868) were observed in participants’ samples. However, no statistically significant differences in the three genes were observed between any CM risk factor in any country (p(fdr-corrected) > 0.05).

**Contributions of CM risk and environmental factors to the inter-individual dissimilarities in oral microbiota composition are diverse between countries**

We next investigated the contribution of each CM risk factor observed in the entire oral microbial dissimilarity matrix (weighted and unweighted UniFrac distance), compared to other participant and lifestyle factors, including gender, age, BMI, sleep hours, smoking and alcohol consumption (**S4 Table**). For weighted UniFrac distance, in the US, CM factors which included waist circumference (R^2^=0.018, p=0.007) and elevated fasting plasma glucose (R^2^=0.013, p=0.036), significantly contributed to the saliva-derived microbiome composition. While, participant and lifestyle factors such as smoking (R^2^=0.071, p=0.001), alcohol consumption (R^2^=0.021, p=0.002) and age (R^2^=0.020, p=0.036) explained a greater proportion of the variance than waist circumference and elevated fasting plasma glucose. In South Africa, only sleep hours (R^2^=0.013, p=0.040), was significantly associated with saliva-derived microbiome composition. In Ghana, gender (R^2^=0.028, p=0.001), sleep hours (R^2^=0.015, p=0.016), alcohol consumption (R^2^=0.011, p=0.049), and Elevated blood pressure (R^2^=0.011, p=0.047), significantly contributed to the saliva-derived microbiome composition. While among Jamaicans, BMI (R^2^=0.069, p=0.011) and age (R^2^=0.060, p=0.006) were significantly associated with the saliva-derived microbiome composition. For the unweighted UniFrac distance, participant and lifestyle factors, including smoking (R^2^=0.077, p=0.001), gender (R^2^= 0.030, p=0.001), age (R^2^=0.019, p=0.031), and alcohol consumption (R^2^=0.016, p=0.008), significantly contributed to the saliva-derived microbiome composition in US population; and CM factors, waist circumference (R^2^=0.016, p=0.009) and HDL concentration (R^2^=0.011, p=0.040), which explained a smaller variance of the saliva-derived microbiome composition. Among South Africans, only waist circumference (R^2^=0.015, p=0.015) was significantly associated with the saliva-derived microbiome composition, which was lower than the other lifestyle factors such as alcohol consumption (R^2^=0.018, p=0.006) and gender (R^2^=0.016, p=0.014). While in Jamaica, lifestyle factors including; smoking (R^2^=0.055, p=0.007), sleep hours (R^2^=0.046, p=0.023), age (R^2^=0.046, p=0.024), gender (R^2^=0.029, p=0.017) and BMI (R^2^=0.029, p=0.021), were significantly associated with saliva-derived microbial composition. However, overall, not one CM risk factor was significantly associated with the saliva-derived microbial composition based on weighted and unweighted UniFrac distance. These results suggest that, like the gut microbiota, both CM risk and lifestyle risk factors contributed significantly to the microbiota composition, in a manner reflective of country of origin.

**Specific oral bacterial taxa associate with individual CM risk factors**

We also used ANCOM to identify specific oral bacterial ESVs that are differentially abundant across groups with the different CM risk factors across the entire cohort (**S17** **Fig** and **S4 Tables**). For these differential ESVs with relative abundance higher than 1% in at least one group, across all the entire cohort (adjusted for country, age, BMI and gender) when stratified by gender (adjusted for country, BMI and age), 3 ESVs (relative abundance ≥ 1% in at least one group) belonging to genus *Streptococcus* (family Streptococcaceae)*, Prevotella* (family Prevotellaceae), and *Veillonella* (family Veillonellaceae) were significantly enriched in individuals with a higher waist circumference, and the same in male and female participants, separately (p(fdr-corrected) <0.05). 1 bacterial ESV annotated to genus *Rothia* (family Micrococcaceae) was significantly enriched in all and female participants with a lower waist circumference. While male participants with a lower waist circumference had significantly enriched 2 bacterial ESVs both annotated to genus *Neisseria* (family Neisseriaceae). 1 bacterial ESV was significantly enriched in people without hyperglyceridemia. 4 bacterial ESVs were significantly enriched in individuals with elevated blood pressure, and the ESVs with relative abundance ≥ 1% in at least one group were annotated to genus *Veillonella* (family Veillonellaceae) (also in male participants), *Prevotella* (family Prevotellaceae) (also in female participants) (p(fdr-corrected) <0.05)*.* 8 bacterial ESVs annotated to genus *Rothia* (family Micrococcaceae, relative abundance ≥ 1% in at least one group, also in female participants)*, Lautropia, Atopobium, Corynebacterium,* *Neisseria* (family Neisseriaceae, relative abundance ≥ 1% in at least one group, also in male participants), family Neisseriaceae, and Pasteurellaceae, separately, were significantly enriched in people without elevated blood pressure (p(fdr-corrected) <0.05). For the HDL concentration, participants with low HDL concentration were enriched with 2 bacterial ESVs annotated to genus *Atopobium*, and family Lachnospiraceae, separately (p(fdr-corrected) <0.05). In male participants with low HDL concentration, 2 bacterial ESVs (relative abundance ≥ 1% in at least one group) annotated to genus *Prevotella* (family Prevotellaceae) and *Neisseria* (family Neisseriaceae) were significantly enriched (p(fdr-corrected) <0.05). 1 bacterial ESV annotated to genus *Streptococcus* was significantly enriched in people without hypertriglyceridemia (p(fdr-corrected) <0.05). Female participants with hypertriglyceridemia enriched with 1 ESV annotated to genus *Veillonella* (family Veillonellaceae), while female participants without hypertriglyceridemia enriched with 1 ESV annotated to genus *Neisseria* (family Neisseriaceae) (p(fdr-corrected) <0.05) (**S17** **Fig** and **S4 Table**). No significantly differential abundant ESVs were found between other pairwise populations (p(fdr-corrected) >0.05).

**Specific oral bacterial taxa are differentially abundant between countries and individual CM risk factors**

Among participants from the US (adjusted for gender, BMI and age), 3 bacterial ESVs (relative abundance ≥ 1% in at least one group) annotated to genus *Neisseria* (family Neisseriaceae)*, Haemophilus* (family Pasteurellaceae)*,* and *Prevotella* (family Prevotellaceae), were significantly enriched in participants with a high waist circumference (p(fdr-corrected) <0.05). 1 bacterial ESV (relative abundance ≥ 1% in at least one group) belonging to genus *Porphyromonas* (family Porphyromonadaceae) was significantly enriched in people with a normal waist circumference (p(fdr-corrected) <0.05). In both South Africans and Ghanaians, 2 bacterial ESVs were significantly enriched in participants with a high waist circumference, and one of them with relative abundance ≥1% in at least one group belongs to genus *Streptococcus* (family Streptococcaceae) (p(fdr-corrected) <0.05). Another 2 bacterial ESVs were significantly enriched in individuals with a normal waist circumference from both South Africa and 1 in Ghana (p(fdr-corrected) <0.05). Among Americans, 1 bacterial ESV with relative abundance ≥1% in at least one group annotated to genus *Haemophilus* was significantly enriched in people without hyperglyceridemia (p(fdr-corrected) <0.05). There was 1 bacterial ESV significantly enriched in participants with Elevated blood pressure in South Africa and Ghana, separately (p(fdr-corrected) <0.05). 2 bacterial ESVs were significantly enriched in participants with low HDL concentration in South Africa (p(fdr-corrected) <0.05). 1 bacterial ESV annotated to genus *Pasteurellaceae* was significantly enriched in participants with hypertriglyceridemia in Ghana (p(fdr-corrected) < 0.05) (**S18** **Fig** and **S2 Table**). No significantly differential abundant ESVs were found between other pairwise populations (p(fdr-corrected) > 0.05).

**Saliva-derived microbiota can predict the gut microbiota by random forest regression**

To test whether the oral microbiota can predict the gut microbiota, random forest regression was used to determine the oral bacterial ESVs against the Shannon index and ESVs (>1%) that were significantly associated with CM risk factors in the gut microbiota (**S4 Table**). Oral microbiota has a trend to predict Shannon index in Ghanaian and Jamaican participants, and the explained variance was 6.8% and 25%, separately. For the differential ESVs in the gut microbiota, the abundance of an ESV assigned to family Clostridiaceae, Peptostreptococcaceae and genus *Prevotella*, separately, could be explained by the saliva microbiota and the explained variance was 7.3%, 7.6% and 6.7% separately. There was a trend for the oral microbiota to predict the ESV assigned to Roseburia (2.6% variance explained).
