# Supplementary figures and images for: The human microbiota is associated with cardiometabolic risk across the epidemiologic transition
Source: PLoS One. 2019 Jul 24;14(7):e0215262. doi: 10.1371/journal.pone.0215262 (PMC6656343; doi:10.1371/journal.pone.0215262)

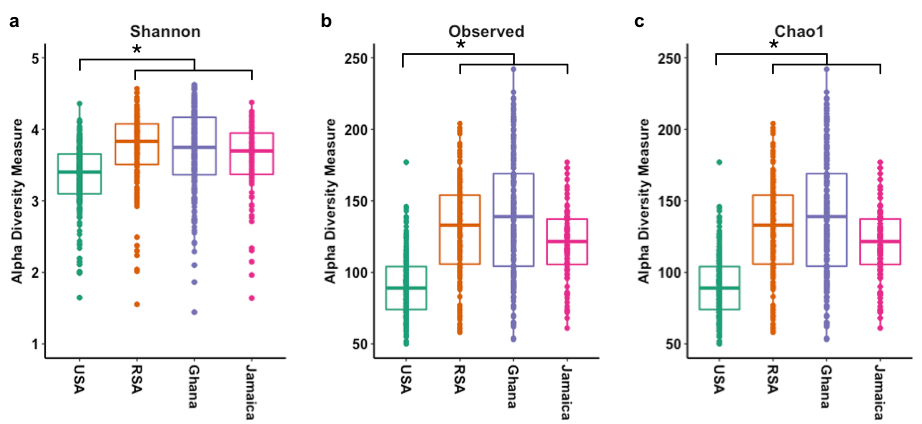

Supplement: S1 Fig — (a-c) Alpha diversity analysis (Shannon Index (a), observed OTU (b) and Chao1 (c)) from 16S rRNA gene sequences of stool microbiota shows the differences among population from USA, RSA, Ghana and Jamaica. USA, the United States of America; RSA, South Africa. * p < 0.05. (TIF) [file pone.0215262.s002.tif]

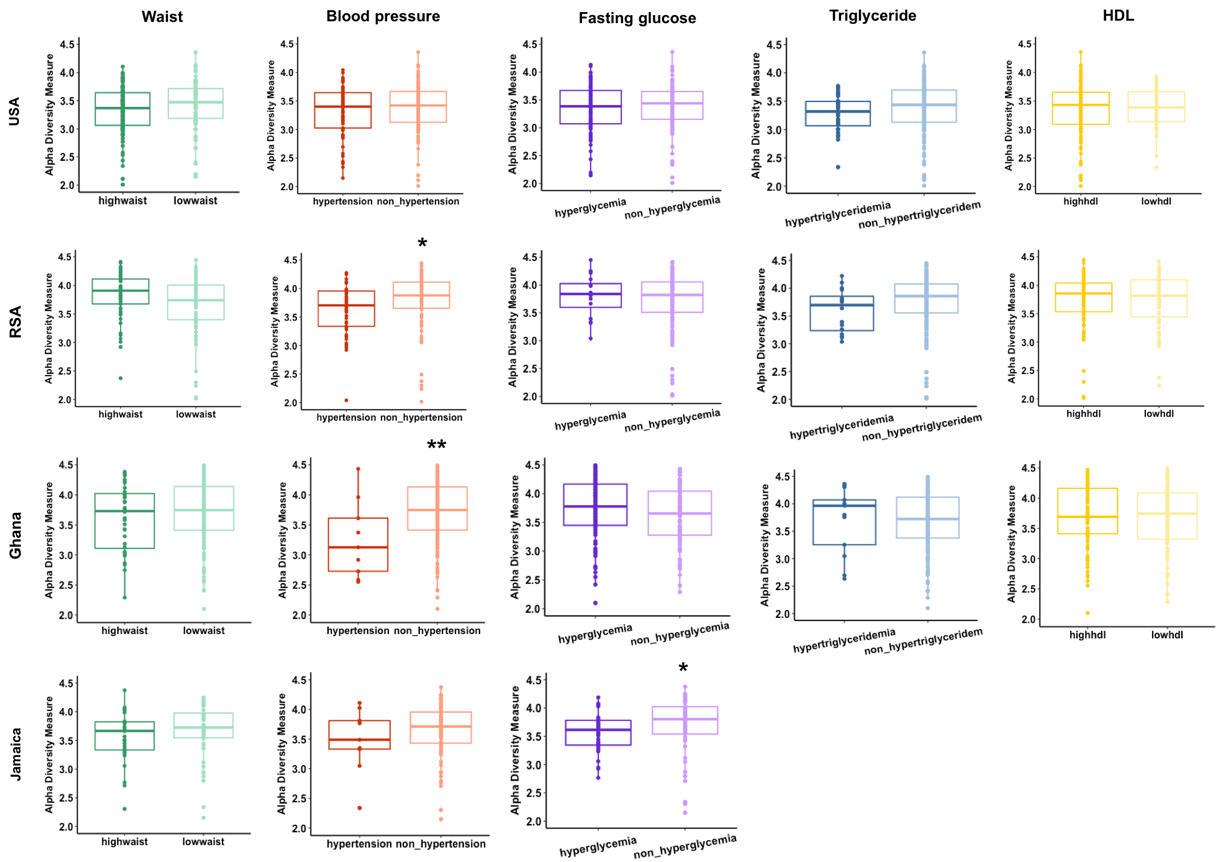

Supplement: S2 Fig — Alpha diversity analysis (Shannon Index) from 16S rRNA gene sequences of stool microbiota against each CM risk factors, including waist circumference, blood pressure, blood fasting glucose, triglyceride and HDL concentration in USA, RSA, Ghanaian and Jamaican populations. USA, the United States of America; RSA, South Africa. * p < 0.05, ** p < 0.01. (TIF) [file pone.0215262.s003.tif]

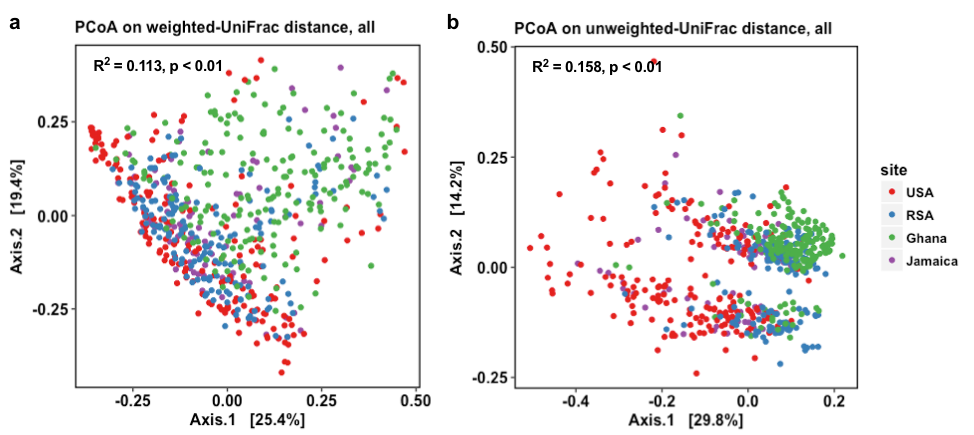

Supplement: S3 Fig — Principal coordinate analyses (PCoA) of weighted (a) and unweighted (b) UniFrac distances of stool microbiota composition in USA, RSA, Ghanaian and Jamaican populations. USA, the United States of America; RSA, South Africa. (TIF) [file pone.0215262.s004.tif]

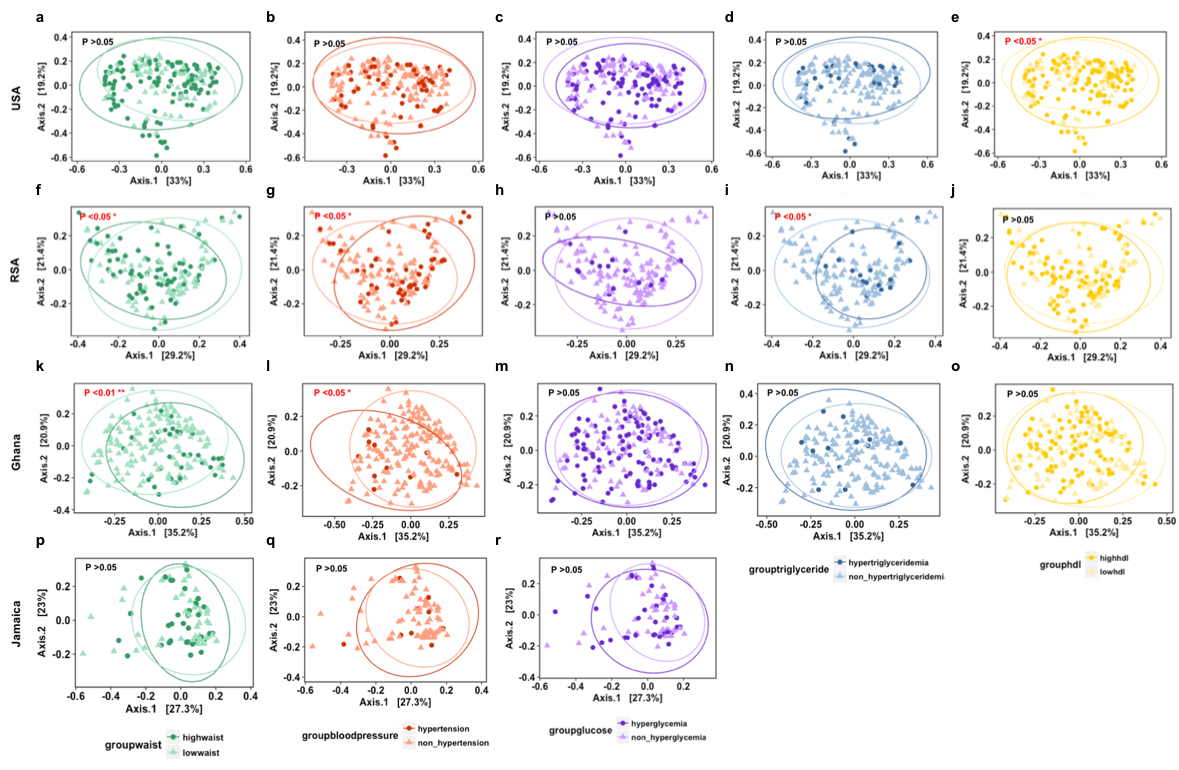

Supplement: S4 Fig — (a-r), Principal coordinate analyses (PCoA) of weighted UniFrac distance of stool microbiota composition against each CM risk factors, including waist circumference, blood pressure, blood fasting glucose, triglyceride and HDL concentration in USA, RSA, Ghanaian and Jamaican populations. USA, the United States of America; RSA, South Africa. (TIF) [file pone.0215262.s005.tif]

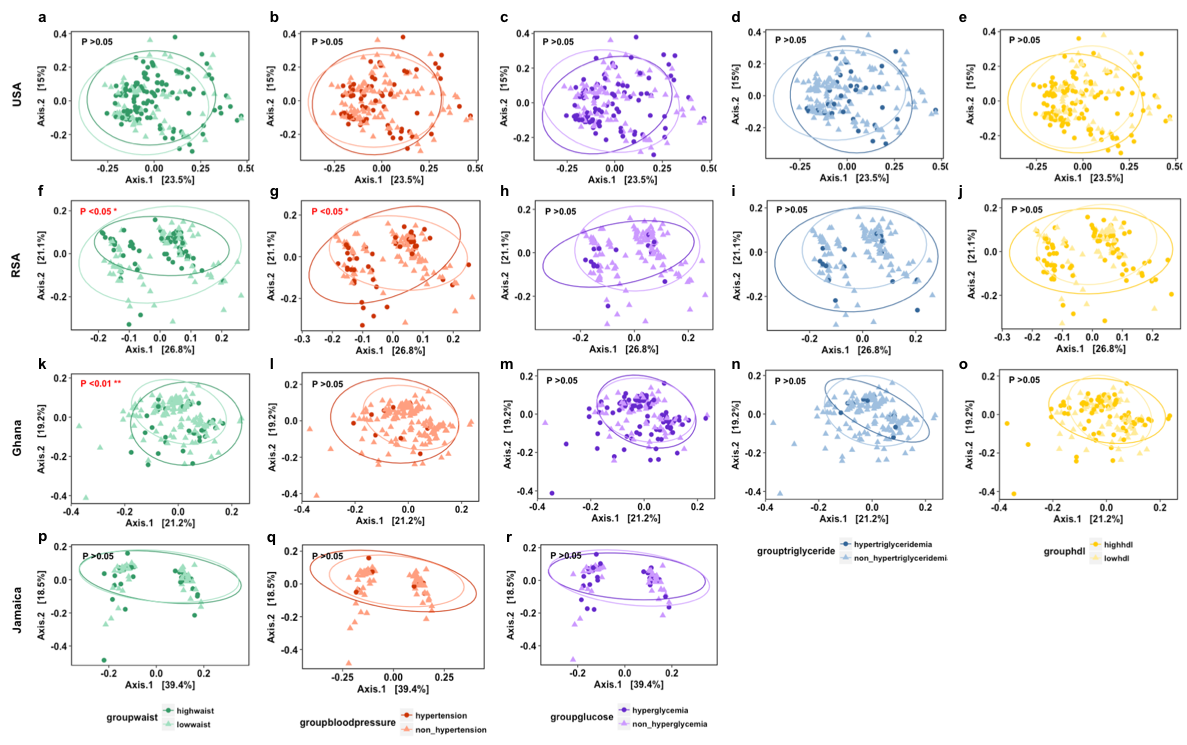

Supplement: S5 Fig — (a-r), Principal coordinate analyses (PCoA) of unweighted UniFrac distance of stool microbiota composition against each CM risk factors, including waist circumference, blood pressure, blood fasting glucose, triglyceride and HDL concentration in USA, RSA, Ghanaian and Jamaican populations. USA, the United States of America; RSA, South Africa. (TIF) [file pone.0215262.s006.tif]

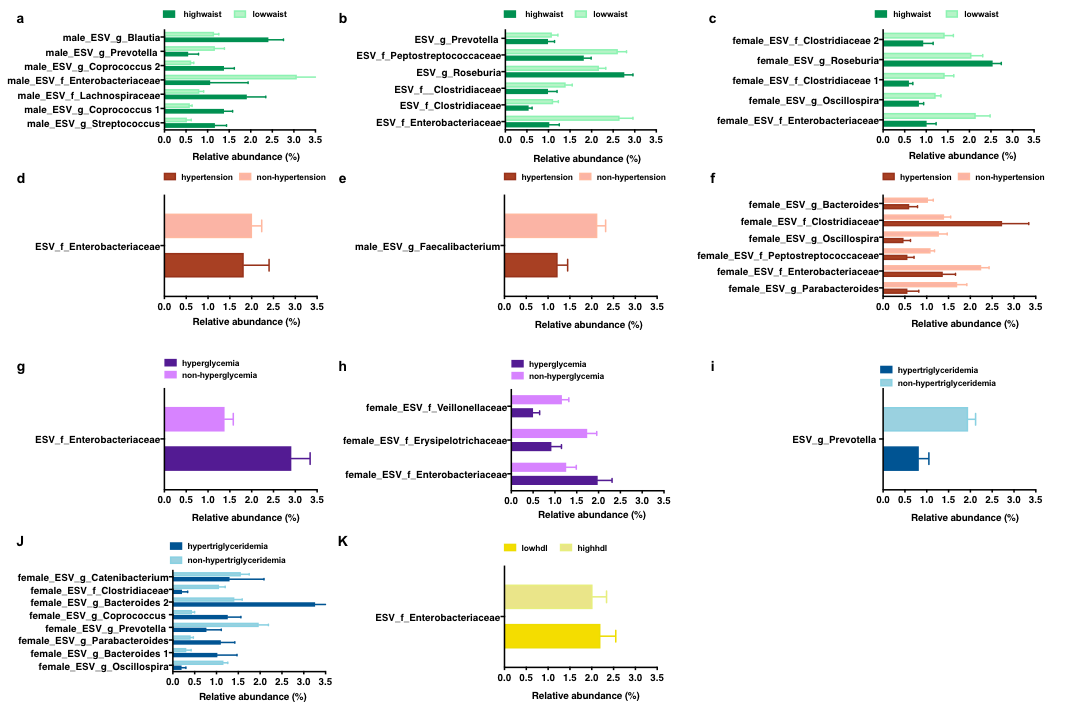

Supplement: S6 Fig — (a-c), Waist circumference across all, male and female participants; (d-f), elevated blood pressure across all, male and female participants; (g-h), elevated fasted blood glucose across all and female participants; (i-j), hypertriglyceridemia across all and female participants; (k), HDL concentration across all and female participants. Only significantly differential ESAs with relative abundance ≥ 1% in at least one group are shown. Data shown are mean± S.E.M. p(fdr-corrected) <0.05. USA, the United States of America; RSA, South Africa. (TIF) [file pone.0215262.s007.tif]

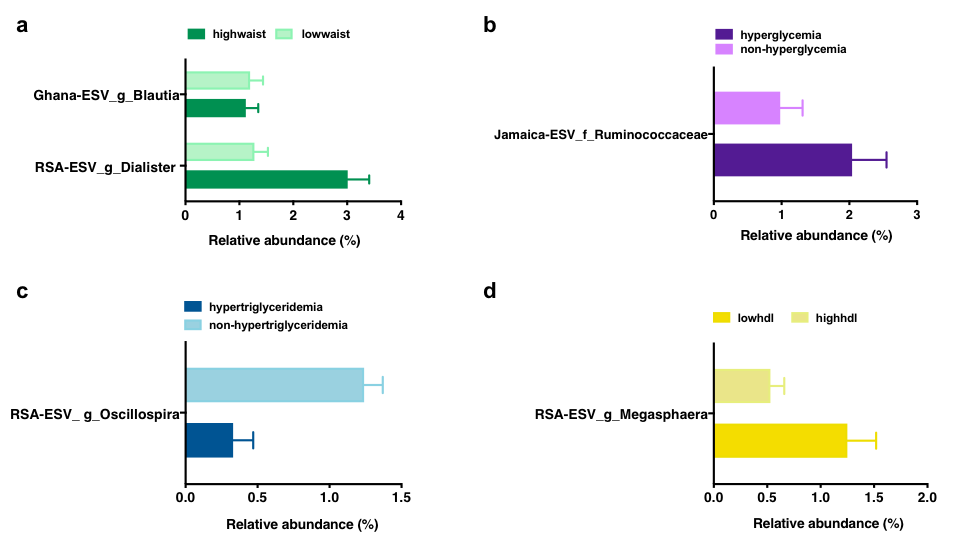

Supplement: S7 Fig — (a), Waist circumference in Ghanaian and RSA population; (b), elevated fasting plasma glucose in Jamaican population; (c), elevated fasting plasma glucose in RSA population; (d), elevated fasting plasma glucose in RSA population. Only significantly differential ESAs with relative abundance ≥ 1% in at least one group are shown. Data shown are mean± S.E.M. p(fdr-corrected) < 0.05. USA, the United States of America; RSA, South Africa. (TIF) [file pone.0215262.s008.tif]

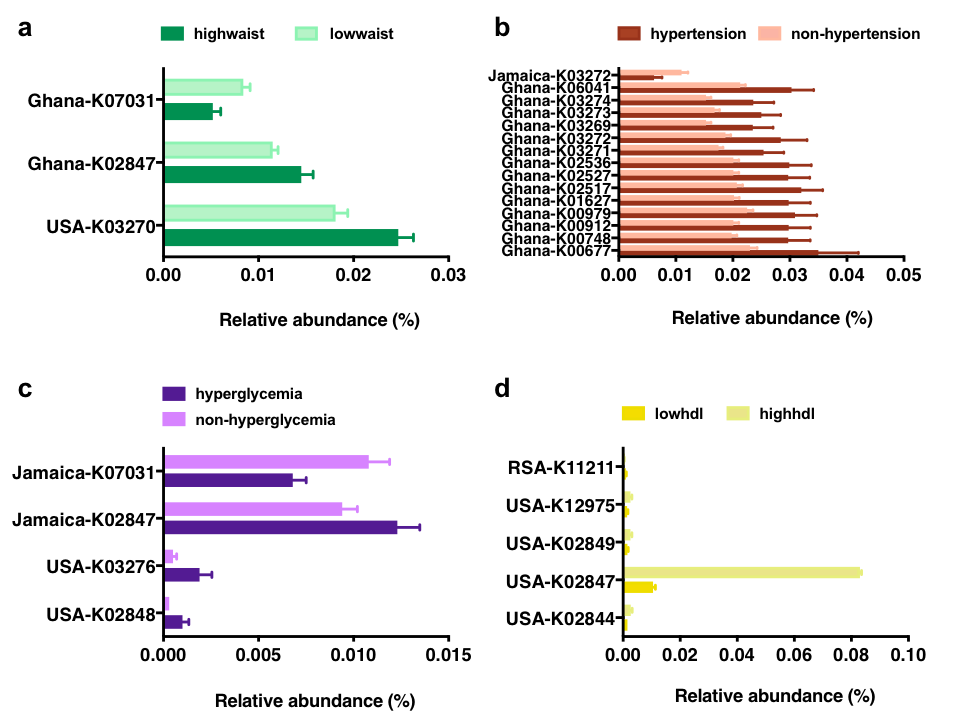

Supplement: S8 Fig — (a), Waist circumference in Ghanaian and USA population; (b), elevated blood pressure in Ghanaian and Jamaican population; (c), elevated fasting plasma glucose in USA and Jamaican population; (d), elevated fasting plasma glucose in USA and RSA population. USA, the United States of America; RSA, South Africa. Only significantly differential KOs are shown. Data shown are mean± S.E.M. p(fdr-corrected) < 0.05. (TIF) [file pone.0215262.s009.tif]

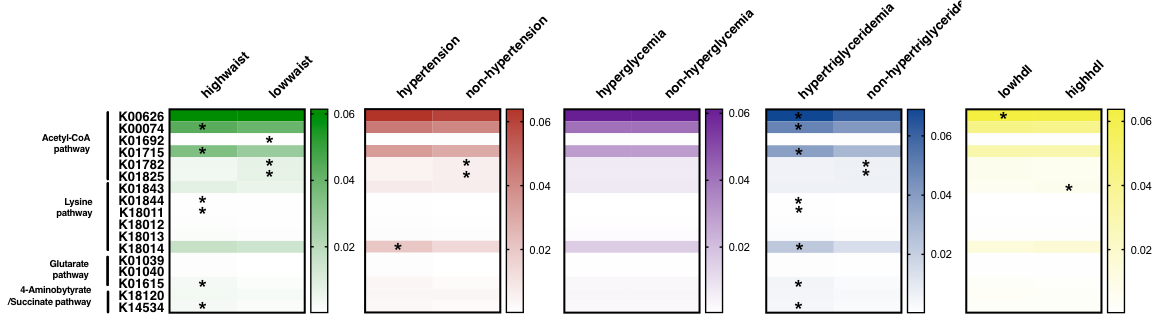

Supplement: S9 Fig — Only significantly differential KOs are shown. Data shown are mean± S.E.M. * p(fdr-corrected) <0.05. (TIF) [file pone.0215262.s010.tif]

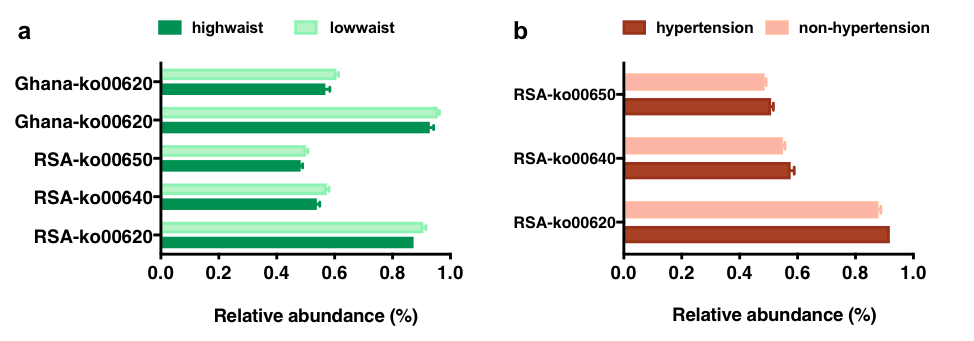

Supplement: S10 Fig — (a), Waist circumference in Ghanaian and RSA population; (b), elevated blood pressure in RSA population. ko00620, pyruvate metabolism, ko00640, propanoate metabolism, and ko00650, butanoate metabolism. USA, the United States of America; RSA, South Africa. Only significantly differential KOs are shown. Data shown are mean± S.E.M. p(fdr-corrected) < 0.05. (TIF) [file pone.0215262.s011.tif]

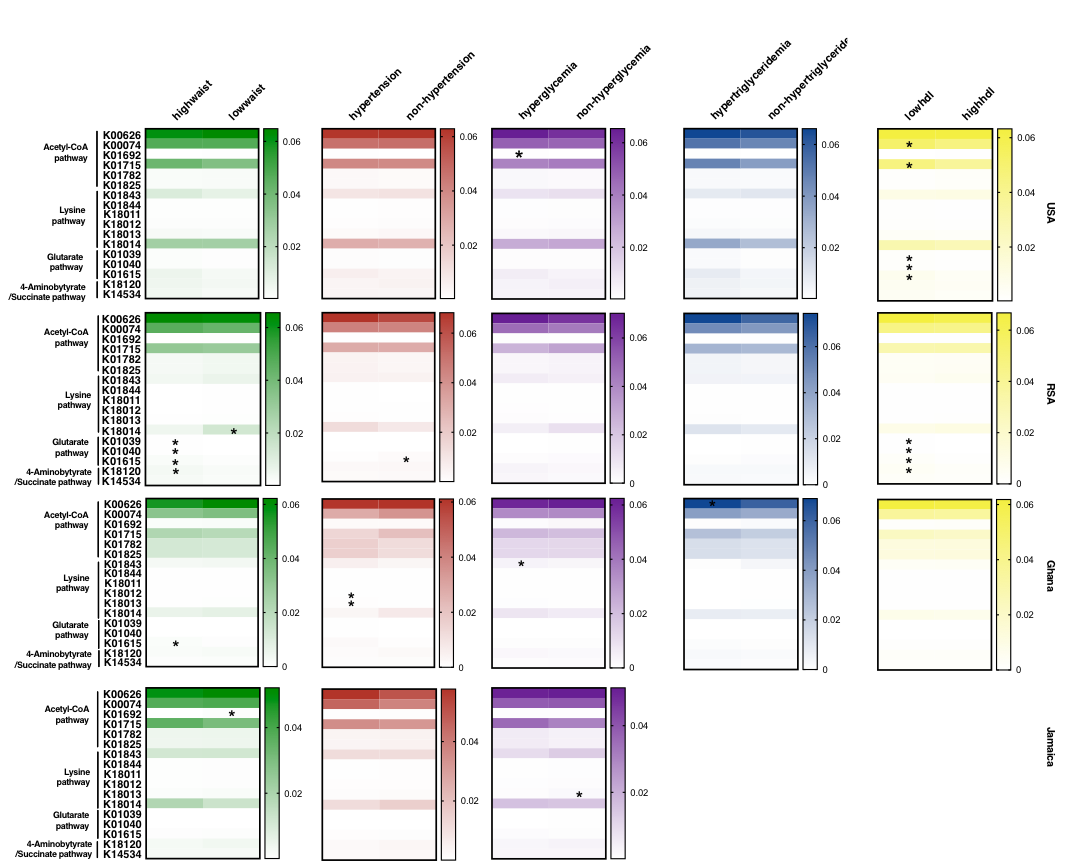

Supplement: S11 Fig — USA, the United States of America; RSA, South Africa. Only significantly differential KOs are shown. Data shown are mean± S.E.M. * p(fdr-corrected) < 0.05. (TIF) [file pone.0215262.s012.tif]

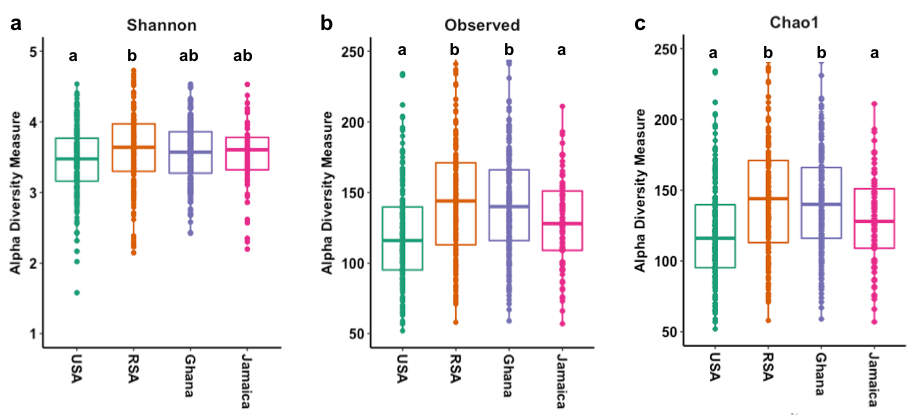

Supplement: S12 Fig — (a-c) Alpha diversity analysis (Shannon Index (a), observed OTU (b) and Chao1 (c)) from 16S rRNA gene sequences from oral microbiota shows the difference among population from USA, RSA, Ghana and Jamaica. USA, the United States of America; RSA, South Africa. No shared letters above the boxplot means p < 0.05 between two groups. (TIF) [file pone.0215262.s013.tif]

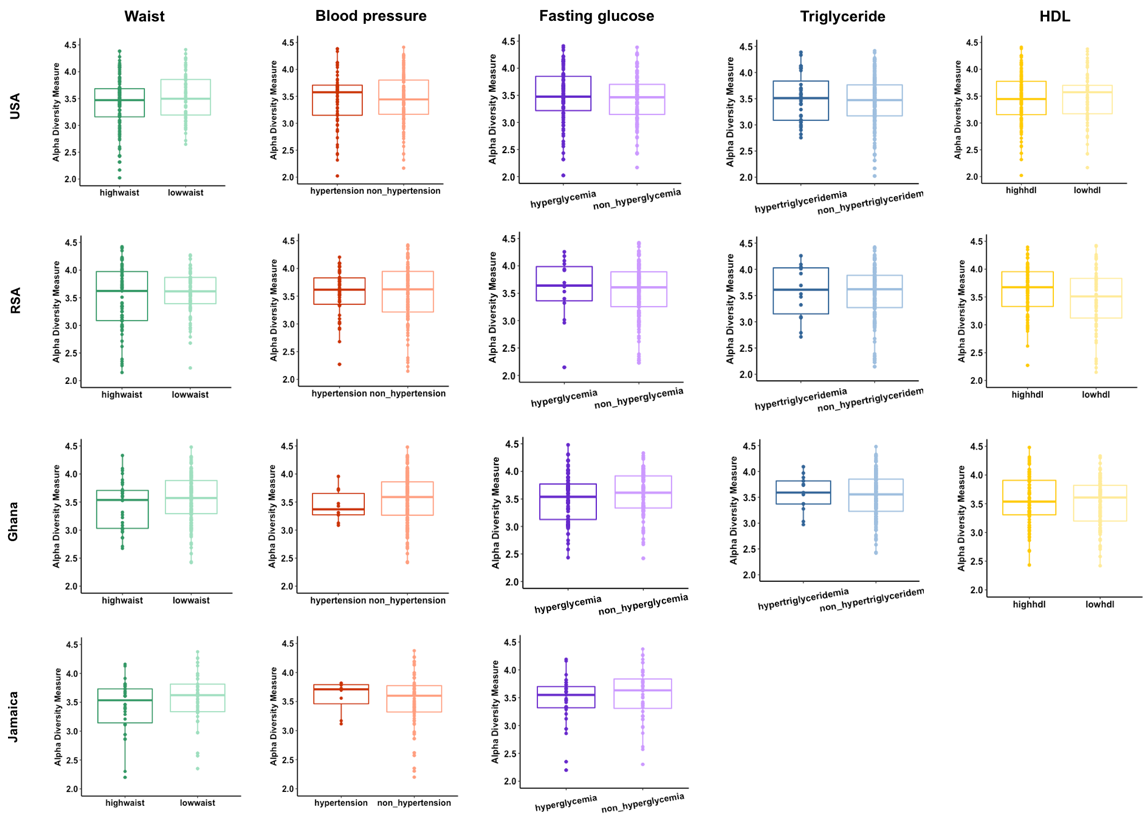

Supplement: S13 Fig — Alpha diversity analysis (Shannon Index) from 16S rRNA gene sequence data of oral microbiota against each CM risk factors, including waist circumference, blood pressure, blood fasting glucose, triglyceride and HDL concentration in USA, RSA, Ghanaian and Jamaican populations. USA, the United States of America; RSA, South Africa. (TIF) [file pone.0215262.s014.tif]

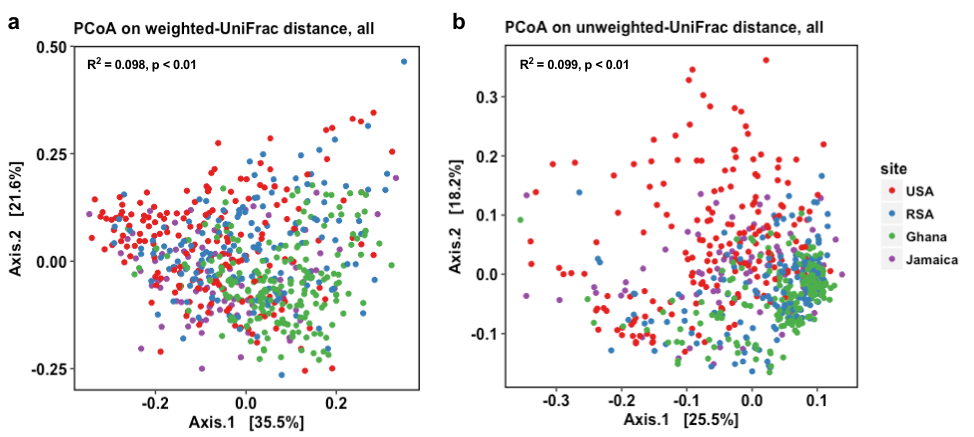

Supplement: S14 Fig — Principal coordinate analyses (PCoA) of weighted (a) and unweighted (b) UniFrac distances of oral microbiota composition in USA, RSA, Ghanaian and Jamaican population. USA, the United States of America; RSA, South Africa. (TIF) [file pone.0215262.s015.tif]

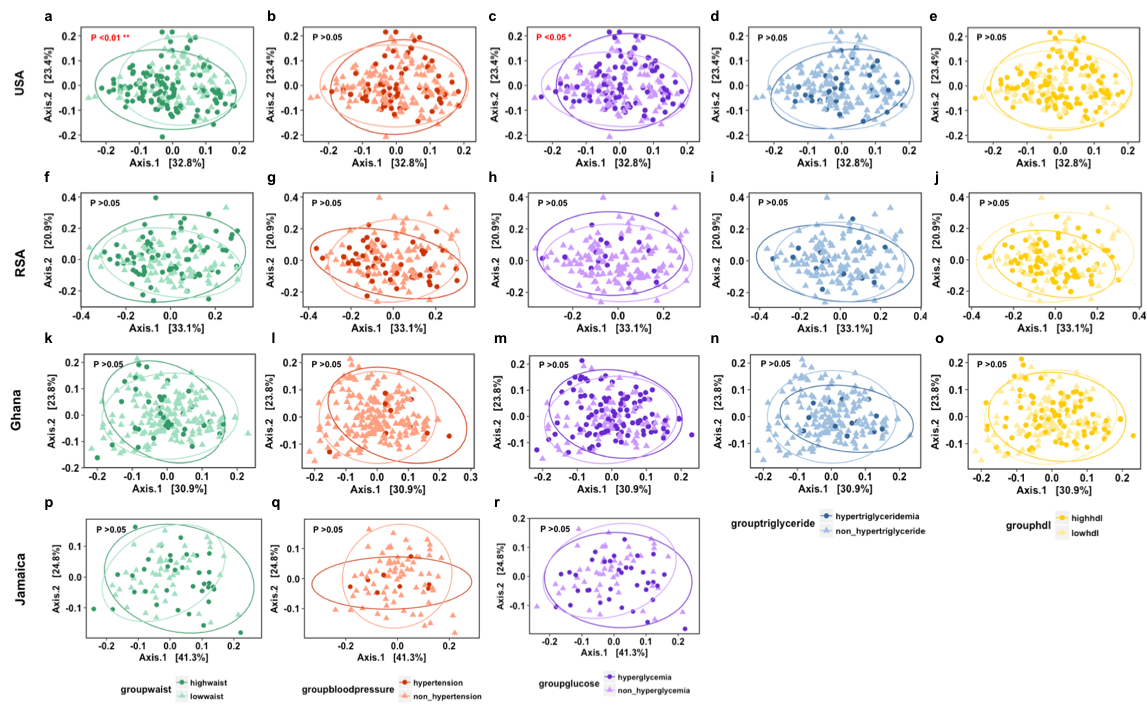

Supplement: S15 Fig — (a-r), Principal coordinate analyses (PCoA) of weighted UniFrac distance of oral microbiota composition against each CM risk factors, including waist circumference, blood pressure, blood fasting glucose, triglyceride and HDL concentration in USA, RSA, Ghanaian and Jamaican populations. USA, the United States of America; RSA, South Africa. (TIF) [file pone.0215262.s016.tif]

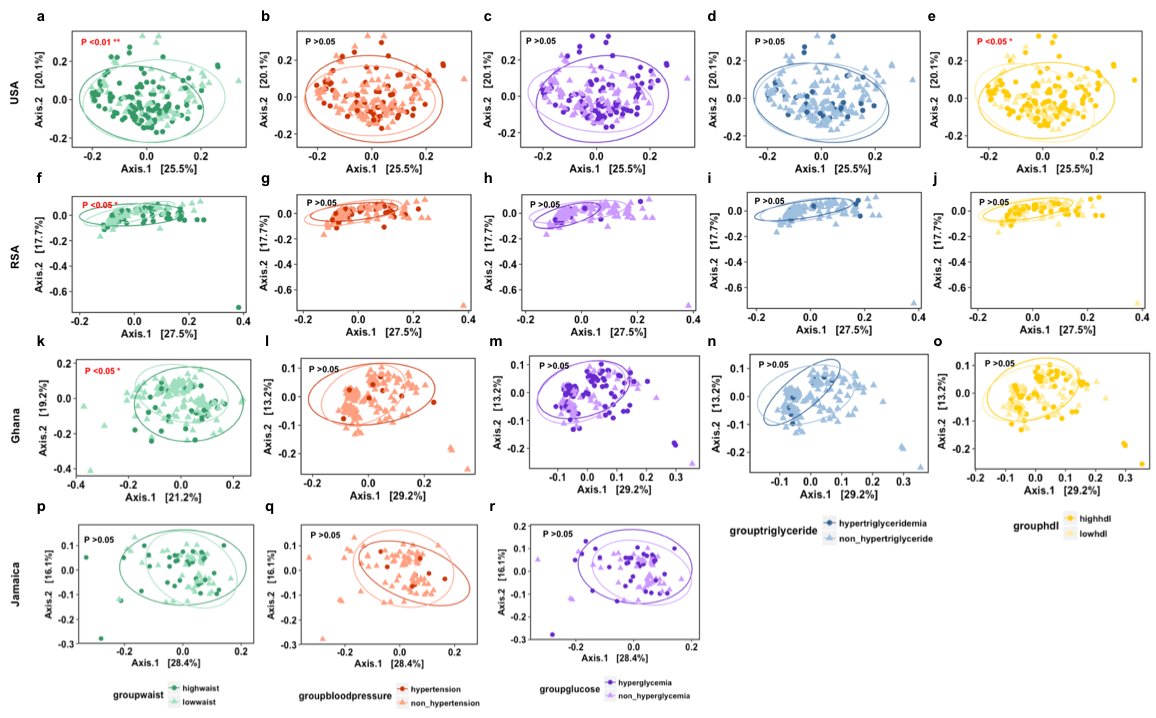

Supplement: S16 Fig — (a-r), Principal coordinate analyses (PCoA) of unweighted UniFrac distance of oral microbiota composition against each CM risk factors, including waist circumference, blood pressure, blood fasting glucose, triglyceride and HDL concentration in USA, RSA, Ghanaian and Jamaican populations. USA, the United States of America; RSA, South Africa. (TIF) [file pone.0215262.s017.tif]

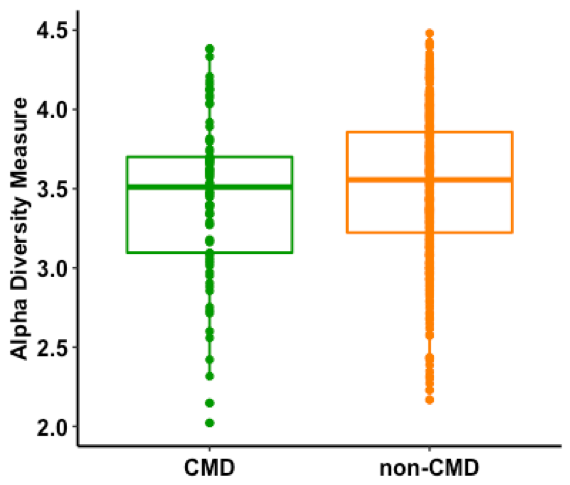

Supplement: S17 Fig — Alpha diversity analysis (Shannon Index) from 16S rRNA gene sequence data of oral microbiota against elevated CM risk, defined as at least of 3 CM risk factors of waist circumference, elevated blood pressure, elevated blood fasting glucose, triglyceride and HDL concentration in USA, RSA, and Ghanaian populations. CMD, elevated CM risk. (TIF) [file pone.0215262.s018.tif]

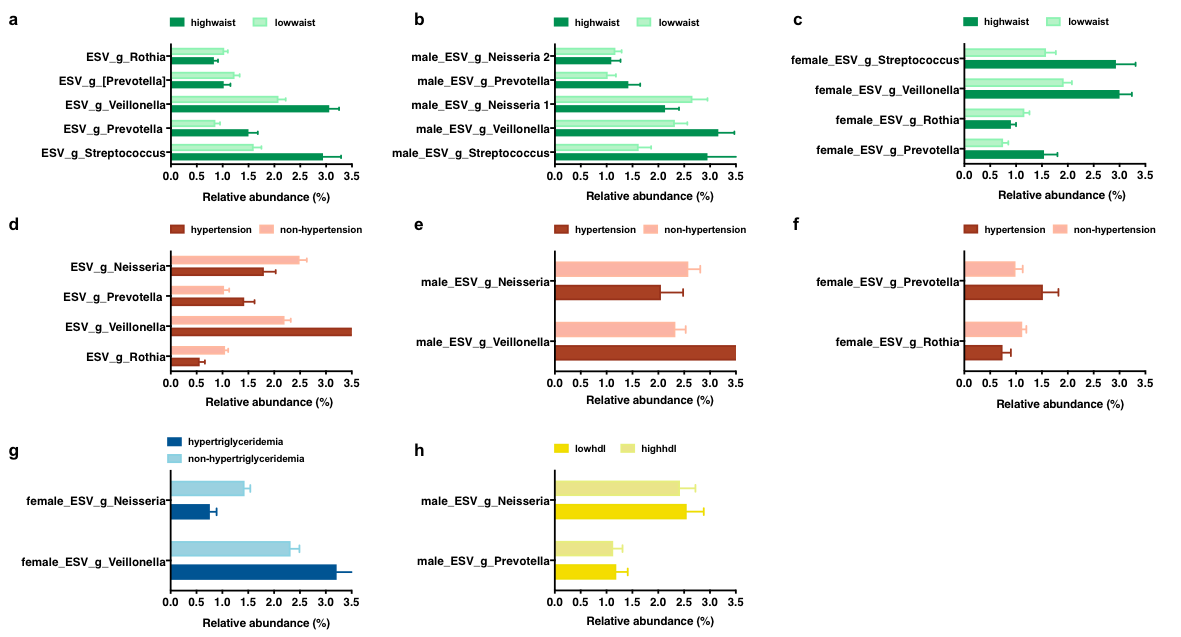

Supplement: S18 Fig — (a-c), Waist circumference across all, male and female participants; (d-f), elevated blood pressure across all, male and female participants; (g), hypertriglyceridemia across female participants; (h) HDL concentration across male participants. Only significantly differential ESAs with relative abundance ≥ 1% in at least one group are shown. Data shown are mean± S.E.M. p(fdr-corrected) <0.05. USA, the United States of America; RSA, South Africa. (TIF) [file pone.0215262.s019.tif]

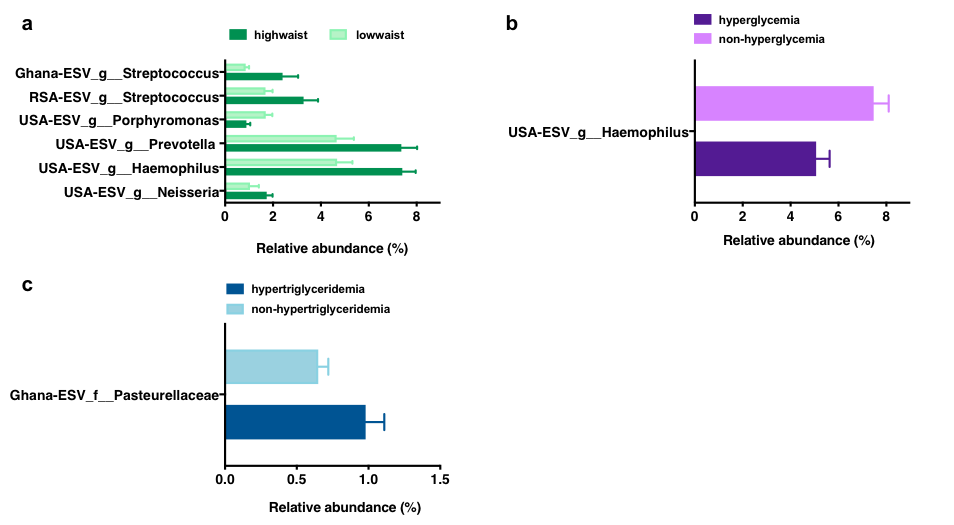

Supplement: S19 Fig — (a), Waist circumference in USA, Ghanaian and RSA population; (b), elevated fasting plasma glucose in USA population; (c), hypertriglyceridemia in Ghanaian population. Only significantly differential ESAs with relative abundance ≥ 1% in at least one group are shown. Data shown are mean± S.E.M. p(fdr-corrected) < 0.05. USA, the United States of America; RSA, South Africa. (TIF) [file pone.0215262.s020.tif]

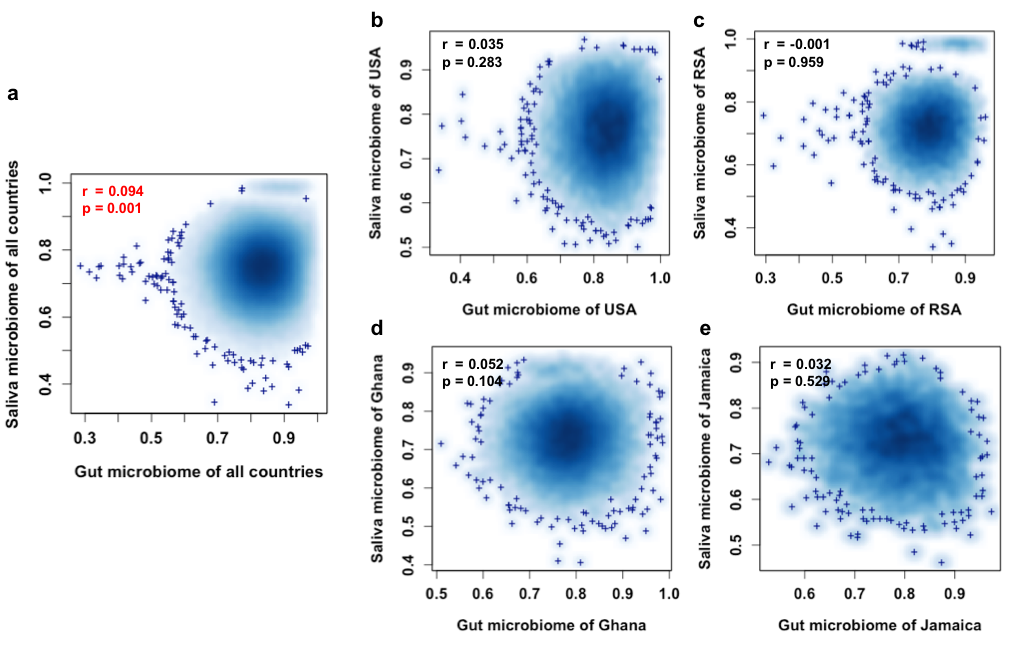

Supplement: S20 Fig — (a) Scatter plot of beta diversity in the gut microbiota versus the oral microbiome independently of the country of origin; (b-e) and for each country (b) USA, (c) RSA, (d), Ghana (e) and Jamaica. USA, the United States of America; RSA, South Africa. (TIF) [file pone.0215262.s021.tif]

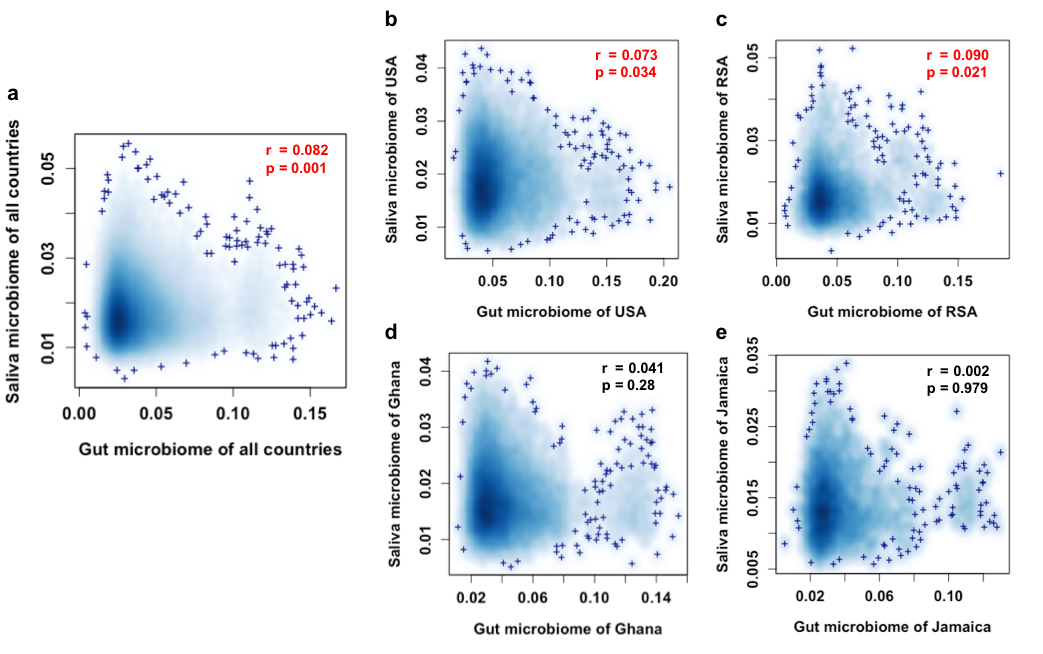

Supplement: S21 Fig — (a) Scatter plot of beta diversity in the gut microbiota versus the oral microbiome independently of the country of origin; (b-e) and for each country (b) USA, (c) RSA, (d), Ghana (e) and Jamaica. USA, the United States of America; RSA, South Africa. (TIF) [file pone.0215262.s022.tif]
