# Supplementary material for: The human microbiota is associated with cardiometabolic risk across the epidemiologic transition
Source: PLoS One. 2019 Jul 24;14(7):e0215262. doi: 10.1371/journal.pone.0215262 (PMC6656343; doi:10.1371/journal.pone.0215262)
Supplement: S1 Table — USA, the United States of America; RSA, South Africa. (DOCX) [file pone.0215262.s023.docx]

**S1 Table.** **Contributions of CM risk factors and participants’ characteristics to the interindividual dissimilarities in gut microbial composition.** USA, the United States of America; RSA, South Africa.

| **Supplementary Table 1a. Gut microbiota (weighted UniFrac distance)** | | | | | | | | | |
| --- | --- | --- | --- | --- | --- | --- | --- | --- | --- |
|  | **USA** | | **RSA** | | **Ghana** | | **Jamaica** | | |
|  | **R^2^** | **P** | **R^2^** | **P** | **R^2^** | **P** | **R^2^** | **P** |  |
| **Waist circumference** | 0.008 | 0.156 | 0.012 | 0.049 | 0.026 | 0.001 | 0.005 | 0.885 |  |
| **Elevated blood pressure** | 0.002 | 0.873 | 0.022 | 0.004 | 0.013 | 0.025 | 0.006 | 0.842 |  |
| **Elevated fasting plasma glucose** | 0.002 | 0.954 | 0.013 | 0.041 | 0.002 | 0.878 | / | / |  |
| **HDL concentration** | 0.013 | 0.030 | 0.009 | 0.153 | 0.006 | 0.275 | / | / |  |
| **Elevated fasting plasma glucose** | 0.008 | 0.166 | 0.005 | 0.543 | 0.003 | 0.789 | 0.010 | 0.522 |  |
| **Gender** | 0.068 | 0.001 | 0.008 | 0.234 | 0.019 | 0.006 | 0.021 | 0.087 |  |
| **Age** | 0.012 | 0.358 | 0.018 | 0.119 | 0.006 | 0.850 | 0.014 | 0.851 |  |
| **BMI** | 0.031 | 0.002 | 0.014 | 0.280 | 0.054 | 0.001 | 0.020 | 0.607 |  |
| **Sleep** | 0.004 | 0.566 | 0.012 | 0.056 | 0.006 | 0.372 | 0.013 | 0.363 |  |
| **Smoke** | 0.032 | 0.011 | 0.016 | 0.657 | 0.020 | 0.049 | 0.044 | 0.567 |  |
| **Alcohol consumption** | 0.017 | 0.010 | 0.004 | 0.731 | 0.013 | 0.039 | 0.010 | 0.509 |  |
|  |  |  |  |  |  |  |  |  |  |
| **Supplementary Table 1b. Gut microbiota (unweighted UniFrac distance)** | | | | | | | | | |
|  | **USA** | | **RSA** | | **Ghana** | | **Jamaica** | | |
|  | **R^2^** | **P** | **R^2^** | **P** | **R^2^** | **P** | **R^2^** | **P** |  |
| **Waist circumference** | 0.008 | 0.145 | 0.014 | 0.034 | 0.019 | 0.001 | 0.007 | 0.675 |  |
| **Elevated blood pressure** | 0.001 | 0.994 | 0.013 | 0.036 | 0.009 | 0.155 | 0.004 | 0.877 |  |
| **Elevated fasting plasma glucose** | 0.006 | 0.409 | 0.009 | 0.234 | 0.002 | 0.871 | / | / |  |
| **HDL concentration** | 0.006 | 0.285 | 0.006 | 0.435 | 0.003 | 0.699 | / | / |  |
| **Elevated fasting plasma glucose** | 0.005 | 0.478 | 0.010 | 0.153 | 0.005 | 0.428 | 0.015 | 0.255 |  |
| **Gender** | 0.045 | 0.001 | 0.008 | 0.240 | 0.015 | 0.011 | 0.019 | 0.122 |  |
| **Age** | 0.014 | 0.165 | 0.018 | 0.128 | 0.001 | 0.999 | 0.017 | 0.701 |  |
| **BMI** | 0.013 | 0.260 | 0.014 | 0.290 | 0.040 | 0.010 | 0.016 | 0.764 |  |
| **Sleep** | 0.008 | 0.138 | 0.012 | 0.076 | 0.007 | 0.277 | 0.007 | 0.709 |  |
| **Smoke** | 0.031 | 0.010 | 0.016 | 0.606 | 0.013 | 0.300 | 0.064 | 0.161 |  |
| **Alcohol consumption** | 0.018 | 0.001 | 0.004 | 0.739 | 0.008 | 0.168 | 0.011 | 0.451 |  |

**Supplementary Table 4. Differential ESV abundance of saliva microbiome by each CM risk including waist circumference, blood pressure, blood fasting glucose, triglyceride and HDL concentration in USA, RSA, Ghanaian and Jamaican population (****adjusted for country, age, BMI and gender across the entire cohort, adjusted for BMI, country and gender in female or male participants, adjusted for BMI, age and gender in each country).** USA, the United States of America; RSA, South Africa. ESV, Exact Sequence Variant. Data shown are mean± S.E.M.

| **Country** | **Taxonomy of Significantly differential ESVs** | **Average relative abundance ***  **(% (SE))** | |
| --- | --- | --- | --- |
| **Group waist** | | **High waist** | **Low waist** |
| **All** | p__Firmicutes; c__Bacilli; o__Lactobacillales; f__Streptococcaceae; g__Streptococcus | 2.94 (0.35) | 1.60 (0.15) |
|  | p__Bacteroidetes; c__Bacteroidia; o__Bacteroidales; f__Prevotellaceae; g__Prevotella | 1.50 (0.18) | 0.86 (0.09) |
|  | p__Firmicutes; c__Clostridia; o__Clostridiales; f__Veillonellaceae; g__Veillonella | 3.06 (0.19) | 2.08 (0.14) |
|  | p__Bacteroidetes; c__Bacteroidia; o__Bacteroidales; f__Prevotellaceae; g__Prevotella | 0.35 (0.04) | 0.64 (0.05) |
|  | p__Bacteroidetes; c__Bacteroidia; o__Bacteroidales; f__Prevotellaceae; g__Prevotella | 0.30 (0.04) | 0.19 (0.03) |
|  | p__Bacteroidetes; c__Bacteroidia; o__Bacteroidales; f__Prevotellaceae; g__Prevotella | 0.13 (0.02) | 0.24 (0.03) |
|  | p__Bacteroidetes; c__Bacteroidia; o__Bacteroidales; f__Prevotellaceae; g__Prevotella | 0.56 (0.04) | 0.44 (0.04) |
|  | p__Firmicutes; c__Clostridia; o__Clostridiales; f__[Tissierellaceae]; g__Parvimonas | 0.13 (0.02) | 0.19 (0.02) |
|  | p__Actinobacteria; c__Actinobacteria; o__Actinomycetales; f__Micrococcaceae; g__Rothia | 0.42 (0.07) | 0.20 (0.04) |
|  | p__Fusobacteria; c__Fusobacteriia; o__Fusobacteriales; f__Leptotrichiaceae; g__ | 0.55 (0.10) | 0.53 (0.06) |
|  | p__Actinobacteria; c__Coriobacteriia; o__Coriobacteriales; f__Coriobacteriaceae; g__Atopobium | 0.17 (0.02) | 0.11 (0.01) |
|  | p__Bacteroidetes; c__Bacteroidia; o__Bacteroidales; f__[Paraprevotellaceae]; g__[Prevotella] | 1.02 (0.13) | 1.23 (0.10) |
|  | p__Firmicutes; c__Clostridia; o__Clostridiales; f__Lachnospiraceae; g__ | 0.06 (0.01) | 0.03 (0.004) |
|  | p__Bacteroidetes; c__Bacteroidia; o__Bacteroidales; f__Prevotellaceae; g__Prevotella | 0.14 (0.01) | 0.21 (0.02) |
|  | p__Proteobacteria; c__Epsilonproteobacteria; o__Campylobacterales; f__Campylobacteraceae; g__Campylobacter | 0.23 (0.01) | 0.20 (0.01) |
|  | p__Bacteroidetes; c__Bacteroidia; o__Bacteroidales; f__Porphyromonadaceae; g__Porphyromonas | 0.47 (0.07) | 0.67 (0.07) |
|  | p__SR1; c__; o__; f__; g__ | 0.06 (0.01) | 0.11 (0.01) |
|  | p__Actinobacteria; c__Actinobacteria; o__Actinomycetales; f__Actinomycetaceae; g__Actinomyces | 0.12 (0.01) | 0.09 (0.01) |
|  | p__Proteobacteria; c__Epsilonproteobacteria; o__Campylobacterales; f__Campylobacteraceae; g__Campylobacter | 0.07 (0.01) | 0.14 (0.02) |
|  | p__Firmicutes; c__Clostridia; o__Clostridiales; f__Peptostreptococcaceae; g__Filifactor | 0.19 (0.02) | 0.26 (0.02) |
|  | p__Bacteroidetes; c__Bacteroidia; o__Bacteroidales; f__[Paraprevotellaceae]; g__[Prevotella] | 0.43 (0.05) | 0.34 (0.04) |
|  | p__Fusobacteria; c__Fusobacteriia; o__Fusobacteriales; f__Fusobacteriaceae; g__Fusobacterium | 0.56 (0.07) | 0.33 (0.03) |
|  | p__Fusobacteria; c__Fusobacteriia; o__Fusobacteriales; f__Fusobacteriaceae; g__Fusobacterium | 0.27 (0.02) | 0.39 (0.03) |
|  | p__Bacteroidetes; c__Bacteroidia; o__Bacteroidales; f__[Paraprevotellaceae]; g__[Prevotella] | 0.24 (0.04) | 0.34 (0.03) |
|  | p__Actinobacteria; c__Actinobacteria; o__Actinomycetales; f__Actinomycetaceae; g__Actinomyces | 0.17 (0.01) | 0.25 (0.01) |
|  | p__Tenericutes; c__Mollicutes; o__Acholeplasmatales; f__Acholeplasmataceae; g__Acholeplasma | 0.07 (0.02) | 0.12 (0.02) |
|  | p__Bacteroidetes; c__Bacteroidia; o__Bacteroidales; f__Prevotellaceae; g__Prevotella | 0.30 (0.04) | 0.17 (0.02) |
|  | p__Firmicutes; c__Clostridia; o__Clostridiales; f__Peptostreptococcaceae; g__Peptostreptococcus | 0.26 (0.02) | 0.37 (0.02) |
|  | p__Firmicutes; c__Clostridia; o__Clostridiales; f__Lachnospiraceae; g__Moryella | 0.08 (0.01) | 0.12 (0.01) |
|  | p__Bacteroidetes; c__Bacteroidia; o__Bacteroidales; f__Porphyromonadaceae; g__Porphyromonas | 0.71 (0.08) | 0.91 (0.07) |
|  | p__Actinobacteria; c__Actinobacteria; o__Actinomycetales; f__Micrococcaceae; g__Rothia | 0.83 (0.08) | 1.03 (0.07) |
| **All_Male** | p__Actinobacteria; c__Coriobacteriia; o__Coriobacteriales; f__Coriobacteriaceae; g__Atopobium | 0.20 (0.03) | 0.16 (0.03) |
|  | p__Firmicutes; c__Bacilli; o__Lactobacillales; f__Streptococcaceae; g__Streptococcus | 2.95 (0.69) | 1.62 (0.24) |
|  | p__Firmicutes; c__Clostridia; o__Clostridiales; f__Veillonellaceae; g__Veillonella | 3.16 (0.31) | 2.32 (0.24) |
|  | p__Actinobacteria; c__Actinobacteria; o__Actinomycetales; f__Micrococcaceae; g__Rothia | 0.21 (0.06) | 0.27 (0.08) |
|  | p__Proteobacteria; c__Betaproteobacteria; o__Neisseriales; f__Neisseriaceae; g__Neisseria | 2.13 (0.27) | 2.66 (0.29) |
|  | p__Fusobacteria; c__Fusobacteriia; o__Fusobacteriales; f__Leptotrichiaceae; g__ | 0.74 (0.23) | 0.43 (0.09) |
|  | p__Bacteroidetes; c__Bacteroidia; o__Bacteroidales; f__Prevotellaceae; g__Prevotella | 1.42 (0.23) | 1.02 (0.16) |
|  | p__Bacteroidetes; c__Bacteroidia; o__Bacteroidales; f__Prevotellaceae; g__Prevotella | 0.20 (0.03) | 0.13 (0.02) |
|  | p__Firmicutes; c__Clostridia; o__Clostridiales; f__[Tissierellaceae]; g__Parvimonas | 0.09 (0.02) | 0.19 (0.02) |
|  | p__Proteobacteria; c__Betaproteobacteria; o__Neisseriales; f__Neisseriaceae; g__Neisseria | 1.09 (0.18) | 1.17 (0.12) |
|  | p__Bacteroidetes; c__Bacteroidia; o__Bacteroidales; f__Prevotellaceae; g__Prevotella | 0.09 (0.02) | 0.25 (0.05) |
|  | p__Fusobacteria; c__Fusobacteriia; o__Fusobacteriales; f__Leptotrichiaceae; g__Leptotrichia | 0.10 (0.03) | 0.07 (0.03) |
|  | p__Bacteroidetes; c__Bacteroidia; o__Bacteroidales; f__Prevotellaceae; g__Prevotella | 0.25 (0.06) | 0.55 (0.12) |
|  | p__Spirochaetes; c__Spirochaetes; o__Spirochaetales; f__Spirochaetaceae; g__Treponema | 0.05 (0.01) | 0.05 (0.02) |
|  | p__Firmicutes; c__Bacilli; o__Lactobacillales; f__Lactobacillaceae; g__Lactobacillus | 0.21 (0.09) | 0.19 (0.10) |
|  | p__Firmicutes; c__Bacilli; o__Lactobacillales; f__Lactobacillaceae; g__Lactobacillus | 0.05 (0.02) | 0.04 (0.02) |
|  | p__SR1; c__; o__; f__; g__ | 0.06 (0.02) | 0.11 (0.02) |
|  | p__Firmicutes; c__Clostridia; o__Clostridiales; f__Lachnospiraceae; g__ | 0.06 (0.01) | 0.04 (0.01) |
|  | p__Bacteroidetes; c__Bacteroidia; o__Bacteroidales; f__[Paraprevotellaceae]; g__[Prevotella] | 0.16 (0.04) | 0.20 (0.02) |
|  | p__Bacteroidetes; c__Bacteroidia; o__Bacteroidales; f__[Paraprevotellaceae]; g__[Prevotella] | 0.82 (0.13) | 1.13 (0.15) |
| **All_Female** | p__Bacteroidetes; c__Bacteroidia; o__Bacteroidales; f__Prevotellaceae; g__Prevotella | 1.54 (0.26) | 0.75 (0.10) |
|  | p__Actinobacteria; c__Actinobacteria; o__Actinomycetales; f__Micrococcaceae; g__Rothia | 0.90 (0.10) | 1.16 (0.10) |
|  | p__Bacteroidetes; c__Bacteroidia; o__Bacteroidales; f__Prevotellaceae; g__Prevotella | 0.37 (0.05) | 0.60 (0.05) |
|  | p__Firmicutes; c__Clostridia; o__Clostridiales; f__Veillonellaceae; g__Veillonella | 3.00 (0.24) | 1.92 (0.16) |
|  | p__Actinobacteria; c__Actinobacteria; o__Actinomycetales; f__Micrococcaceae; g__Rothia | 0.47 (0.09) | 0.19 (0.06) |
|  | p__Actinobacteria; c__Coriobacteriia; o__Coriobacteriales; f__Coriobacteriaceae; g__Atopobium | 0.16 (0.02) | 0.08 (0.01) |
|  | p__Fusobacteria; c__Fusobacteriia; o__Fusobacteriales; f__Leptotrichiaceae; g__ | 0.45 (0.10) | 0.60 (0.07) |
|  | p__Bacteroidetes; c__Bacteroidia; o__Bacteroidales; f__Prevotellaceae; g__Prevotella | 0.32 (0.06) | 0.17 (0.04) |
|  | p__Firmicutes; c__Bacilli; o__Lactobacillales; f__Streptococcaceae; g__Streptococcus | 2.93 (0.38) | 1.58 (0.19) |
|  | p__Actinobacteria; c__Actinobacteria; o__Actinomycetales; f__Actinomycetaceae; g__Actinomyces | 0.18 (0.02) | 0.25 (0.02) |
|  | p__Bacteroidetes; c__Bacteroidia; o__Bacteroidales; f__[Paraprevotellaceae]; g__[Prevotella] | 0.24 (0.05) | 0.35 (0.04) |
|  | p__Bacteroidetes; c__Bacteroidia; o__Bacteroidales; f__Prevotellaceae; g__Prevotella | 0.15 (0.03) | 0.24 (0.03) |
|  | p__Firmicutes; c__Clostridia; o__Clostridiales; f__[Tissierellaceae]; g__Parvimonas | 0.15 (0.03) | 0.19 (0.02) |
|  | p__Fusobacteria; c__Fusobacteriia; o__Fusobacteriales; f__Fusobacteriaceae; g__Fusobacterium | 0.64 (0.11) | 0.32 (0.04) |
|  | p__Actinobacteria; c__Coriobacteriia; o__Coriobacteriales; f__Coriobacteriaceae; g__Atopobium | 0.08 (0.02) | 0.09 (0.02) |
|  | p__Bacteroidetes; c__Bacteroidia; o__Bacteroidales; f__Prevotellaceae; g__Prevotella | 0.52 (0.05) | 0.40 (0.04) |
|  | p__Bacteroidetes; c__Bacteroidia; o__Bacteroidales; f__Prevotellaceae; g__Prevotella | 0.15 (0.02) | 0.20 (0.02) |
|  | p__Bacteroidetes; c__Bacteroidia; o__Bacteroidales; f__[Paraprevotellaceae]; g__[Prevotella] | 0.37 (0.05) | 0.29 (0.04) |
|  | p__Firmicutes; c__Clostridia; o__Clostridiales; f__Peptostreptococcaceae; g__Peptostreptococcus | 0.30 (0.03) | 0.40 (0.03) |
|  | p__Firmicutes; c__Clostridia; o__Clostridiales; f__Lachnospiraceae; g__Moryella | 0.09 (0.01) | 0.12 (0.01) |
|  | p__Bacteroidetes; c__Bacteroidia; o__Bacteroidales; f__[Paraprevotellaceae]; g__[Prevotella] | 1.13 (0.19) | 1.30 (0.13) |
| **USA** | p__Proteobacteria; c__Betaproteobacteria; o__Neisseriales; f__Neisseriaceae; g__Neisseria | 1.74 (0.24) | 1.04 (0.37) |
|  | p__Proteobacteria; c__Gammaproteobacteria; o__Pasteurellales; f__Pasteurellaceae; g__Haemophilus | 7.40 (0.56) | 4.68 (0.64) |
|  | p__Bacteroidetes; c__Bacteroidia; o__Bacteroidales; f__Prevotellaceae; g__Prevotella | 7.36 (0.66) | 4.66 (0.72) |
|  | p__Bacteroidetes; c__Bacteroidia; o__Bacteroidales; f__Porphyromonadaceae; g__Porphyromonas | 0.89 (0.16) | 1.70 (0.27) |
| **RSA** | p__Proteobacteria; c__Epsilonproteobacteria; o__Campylobacterales; f__Campylobacteraceae; g__Campylobacter | 0.07 (0.02) | 0.17 (0.03) |
|  | p__Actinobacteria; c__Actinobacteria; o__Actinomycetales; f__Actinomycetaceae; g__Actinomyces | 0.11 (0.02) | 0.21 (0.02) |
|  | p__Firmicutes; c__Bacilli; o__Lactobacillales; f__Streptococcaceae; g__Streptococcus | 3.28 (0.60) | 1.692 (0.29) |
|  | p__Actinobacteria; c__Actinobacteria; o__Actinomycetales; f__Actinomycetaceae; g__Actinomyces | 0.18 (0.02) | 0.12 (0.02) |
| **Ghana** | p__Firmicutes; c__Bacilli; o__Lactobacillales; f__Streptococcaceae; g__Streptococcus | 2.40 (0.65) | 0.86 (0.14) |
|  | p__Bacteroidetes; c__Bacteroidia; o__Bacteroidales; f__Prevotellaceae; g__Prevotella | 0.09 (0.03) | 0.20 (0.02) |
|  | p__Bacteroidetes; c__Bacteroidia; o__Bacteroidales; f__Prevotellaceae; g__Prevotella | 0.26 (0.07) | 0.22 (0.05) |
| **Group glucose** | | **Elevated fasting plasma glucose** | **Non- elevated fasting plasma glucose** |
| **All** | p__Actinobacteria; c__Actinobacteria; o__Actinomycetales; f__Micrococcaceae; g__Rothia | 0.17 (0.04) | 0.37 (0.05) |
| **All_Male** | p__Firmicutes; c__Bacilli; o__Lactobacillales; f__Streptococcaceae; g__Streptococcus | 0.05 (0.01) | 0.05 (0.01) |
|  | p__Firmicutes; c__Bacillio__Lactobacillales; f__Carnobacteriaceae; g__Granulicatella | 0.57 (0.10) | 0.83 (0.09) |
| **All_Female** | p__Actinobacteria; c__Coriobacteriia; o__Coriobacteriales; f__Coriobacteriaceae; g__Atopobium | 0.10 (0.02) | 0.08 (0.01) |
|  | p__Actinobacteria; c__Actinobacteria; o__Actinomycetales; f__Micrococcaceae; g__Rothia | 0.16 (0.04) | 0.39 (0.08) |
| **USA** | p__Proteobacteria; c__Gammaproteobacteria; o__Pasteurellales; f__Pasteurellaceae; g__Haemophilus | 5.07 (0.56) | 7.47 (0.63) |
| **Group blood pressure** | | **Elevated blood pressure** | **Non- Elevated blood pressure** |
| **All** | p__Actinobacteria; c__Coriobacteriia; o__Coriobacteriales; f__Coriobacteriaceae; g__Atopobium | 0.22 (0.03) | 0.11 (0.01) |
|  | p__Actinobacteria; c__Actinobacteria; o__Actinomycetales; f__Micrococcaceae; g__Rothia | 0.56 (0.10) | 1.05 (0.06) |
|  | p__Firmicutes; c__Clostridia; o__Clostridiales; f__Veillonellaceae; g__Veillonella | 3.52 (0.29) | 2.20 (0.12) |
|  | p__Actinobacteria; c__Actinobacteria; o__Actinomycetales; f__Micrococcaceae; g__Rothia | 0.13 (0.02) | 0.30 (0.03) |
|  | p__Bacteroidetes; c__Bacteroidia; o__Bacteroidales; f__Prevotellaceae; g__Prevotella | 1.46 (0.20) | 1.03 (0.10) |
|  | p__Proteobacteria; c__Betaproteobacteria; o__Burkholderiales; f__Burkholderiaceae; g__Lautropia | 0.16 (0.02) | 0.47 (0.05) |
|  | p__Actinobacteria; c__Coriobacteriia; o__Coriobacteriales; f__Coriobacteriaceae; g__Atopobium | 0.04 (0.01) | 0.09 (0.01) |
|  | p__Fusobacteria; c__Fusobacteriia; o__Fusobacteriales; f__Fusobacteriaceae; g__Fusobacterium | 0.13 (0.02) | 0.07 (0.01) |
|  | p__Actinobacteria; c__Actinobacteria; o__Actinomycetales; f__Corynebacteriaceae; g__Corynebacterium | 0.03 (0.004) | 0.07 (0.005) |
|  | p__Proteobacteria; c__Betaproteobacteria; o__Neisseriales; f__Neisseriaceae; g__Neisseria | 1.80 (0.23) | 2.49 (0.14) |
|  | p__Proteobacteria; c__Betaproteobacteria; o__Neisseriales; f__Neisseriaceae; g__ | 0.01 (0.01) | 0.07 (0.01) |
|  | p__Proteobacteria; c__Gammaproteobacteria; o__Pasteurellales; f__Pasteurellaceae | 0.27 (0.04) | 0.50 (0.05) |
| **All_Male** | p__Proteobacteria; c__Gammaproteobacteria; o__Pasteurellales; f__Pasteurellaceae | 0.25 (0.06) | 0.45 (0.08) |
|  | p__Firmicutes; c__Clostridia; o__Clostridiales; f__Veillonellaceae; g__Veillonella | 3.61 (0.44) | 2.33 (0.20) |
|  | p__Proteobacteria; c__Betaproteobacteria; o__Neisseriales; f__Neisseriaceae; g__Neisseria | 2.05 (0.43) | 2.58 (0.23) |
|  | p__Actinobacteria; c__Coriobacteriia; o__Coriobacteriales; f__Coriobacteriaceae; g__Atopobium | 0.26 (0.05) | 0.15 (0.02) |
|  | p__Actinobacteria; c__Actinobacteria; o__Actinomycetales; f__Micrococcaceae; g__Rothia | 0.34 (0.09) | 0.92 (0.10) |
|  | p__Proteobacteria; c__Betaproteobacteria; o__Burkholderiales; f__Burkholderiaceae; g__Lautropia | 0.19 (0.05) | 0.48 (0.11) |
| **All_Female** | p__Actinobacteria; c__Coriobacteriia; o__Coriobacteriales; f__Coriobacteriaceae; g__Atopobium | 0.19 (0.04) | 0.09 (0.01) |
|  | p__Actinobacteria; c__Actinobacteria; o__Actinomycetales; f__Micrococcaceae; g__Rothia | 0.74 (0.16) | 1.12 (0.08) |
|  | p__Bacteroidetes; c__Bacteroidia; o__Bacteroidales; f__Prevotellaceae; g__Prevotella | 1.52 (0.30) | 0.99 (0.14) |
|  | p__Actinobacteria; c__Actinobacteria; o__Actinomycetales; f__Micrococcaceae; g__Rothia | 0.16 (0.03) | 0.28 (0.04) |
|  | p__Bacteroidetes; c__Bacteroidia; o__Bacteroidales; f__[Paraprevotellaceae]; g__[Prevotella] | 0.14 (0.03) | 0.17 (0.02) |
| **RSA** | p__Fusobacteria; c__Fusobacteriia; o__Fusobacteriales; f__Fusobacteriaceae; g__Fusobacterium | 0.15 (0.03) | 0.05 (0.01) |
| **Ghana** | p__Firmicutes; c__Clostridia; o__Clostridiales; f__Veillonellaceae; g__Veillonella | 0.19 (0.08) | 0.04 (0.01) |
| **Group HDL** | | **Low HDL** | **High HDL** |
| **All** | p__Actinobacteria; c__Coriobacteriia; o__Coriobacteriales; f__Coriobacteriaceae; g__Atopobium | 0.09 (0.01) | 0.05 (0.01) |
|  | p__Firmicutes; c__Clostridia; o__Clostridiales; f__Lachnospiraceae | 0.12 (0.01) | 0.09 (0.01) |
| **All_Male** | p__Bacteroidetes; c__Bacteroidia; o__Bacteroidales; f__Prevotellaceae; g__Prevotella | 1.19 (0.22) | 1.13 (0.18) |
|  | p__Proteobacteria; c__Betaproteobacteria; o__Neisseriales; f__Neisseriaceae; g__Neisseria | 2.55 (0.33) | 2.42 (0.30) |
| **RSA** | p__Actinobacteria; c__Actinobacteria; o__Actinomycetales; f__Micrococcaceae; g__Rothia | 0.23 (0.04) | 0.14 (0.03) |
|  | p__Firmicutes; c__Bacilli; o__Lactobacillales; f__Streptococcaceae; g__Streptococcus | 0.67 (0.11) | 0.35 (0.06) |
| **Group triglyceride** | | **Hyper-triglyceridemia** | **Non-hyper-triglyceridemia** |
| **All** | p__Firmicutes; c__Bacilli; o__Lactobacillales; f__Streptococcaceae; g__Streptococcus | 0.58 (0.14) | 0.66 (0.04) |
| **All_Female** | p__Firmicutes; c__Clostridia; o__Clostridiales; f__Veillonellaceae; g__Veillonella | 3.21 (0.55) | 2.32 (0.17) |
|  | p__Firmicutes; c__Bacilli; o__Lactobacillales; f__Streptococcaceae; g__Streptococcus | 0.63 (0.19) | 0.70 (0.06) |
|  | p__Fusobacteria; c__Fusobacteriia; o__Fusobacteriales; f__Fusobacteriaceae; g__Fusobacterium | 0.48 (0.11) | 0.64 (0.04) |
|  | p__Proteobacteria; c__Betaproteobacteria; o__Neisseriales; f__Neisseriaceae; g__Neisseria | 0.76 (0.13) | 1.43 (0.11) |
|  | p__Bacteroidetes; c__Bacteroidia; o__Bacteroidales; f__[Paraprevotellaceae]; g__[Prevotella] | 0.14 (0.04) | 0.18 (0.02) |
|  | p__Bacteroidetes; c__Bacteroidia; o__Bacteroidales; f__Prevotellaceae; g__Prevotella | 0.35 (0.12) | 0.23 (0.04) |
| **Ghana** | p__Proteobacteria; c__Gammaproteobacteria; o__Pasteurellales; f__Pasteurellaceae | 0.98 (0.13) | 0.65 (0.07) |

*Number in red, ESVs were significantly enriched in patients with one of the CM risk factors.
