# Supplementary material for: The human microbiota is associated with cardiometabolic risk across the epidemiologic transition
Source: PLoS One. 2019 Jul 24;14(7):e0215262. doi: 10.1371/journal.pone.0215262 (PMC6656343; doi:10.1371/journal.pone.0215262)
Supplement: S2 Table — USA, the United States of America; RSA, South Africa. ESV, Exact Sequence Variant. Data shown are mean± S.E.M. (DOCX) [file pone.0215262.s024.docx]

**S2 Table.** **Differential ESV abundance of gut microbiota by each CM risk including waist circumference, blood pressure, blood fasting glucose, triglyceride and HDL concentration in USA, RSA, Ghanaian and Jamaican** **population (adjusted for country, age, BMI and gender across the entire cohort,).** USA, the United States of America; RSA, South Africa. ESV, Exact Sequence Variant. Data shown are mean± S.E.M.

| **Country** | **Taxonomy of Significantly differential ESVs** | **Average relative abundance ***  **(% (SE))** | |
| --- | --- | --- | --- |
| **Group waist** | | **High waist** | **Low waist** |
| **All** | p__Proteobacteria; c__Gammaproteobacteria; o__Enterobacteriales; f__Enterobacteriaceae | 1.02 (0.23) | 2.65 (0.31) |
|  | p__Firmicutes; c__Clostridia; o__Clostridiales; f__Clostridiaceae;  g__; | 0.54 (0.08) | 1.11 (0.12) |
|  | p__Firmicutes; c__Erysipelotrichi; o__Erysipelotrichales; f__Erysipelotrichaceae; g__ | 0.51 (0.08) | 0.67 (0.07) |
|  | p__Firmicutes; c__Bacilli; o__Lactobacillales; f__Leuconostocaceae;  g__ | 0.07 (0.03) | 0.27 (0.07) |
|  | p__Firmicutes; c__Clostridia; o__Clostridiales; f__Clostridiaceae;  g__; | 0.99 (0.21) | 1.40 (0.15) |
|  | p__Firmicutes; c__Clostridia; o__Clostridiales; f__Clostridiaceae;  g__SMB53 | 0.23 (0.031) | 0.38 (0.04) |
|  | p__Firmicutes; c__Clostridia; o__Clostridiales; f__Veillonellaceae;  g__Megasphaera | 0.57 (0.11) | 0.30 (0.06) |
|  | p__Firmicutes; c__Bacilli; o__Lactobacillales; f__Streptococcaceae;  g__Streptococcus | 0.07 (0.03) | 0.42 (0.10) |
|  | p__Firmicutes; c__Bacilli; o__Turicibacterales; f__Turicibacteraceae;  g__Turicibacter | 0.21 (0.04) | 0.28 (0.03) |
|  | p__Firmicutes; c__Bacilli; o__Lactobacillales; f__Streptococcaceae;  g__Streptococcus | 0.71 (0.10) | 0.43 (0.06) |
|  | p__Bacteroidetes; c__Bacteroidia; o__Bacteroidales; f__Bacteroidaceae; g__Bacteroides | 0.70 (0.14) | 0.56 (0.13) |
|  | p__Firmicutes; c__Clostridia; o__Clostridiales; f__Ruminococcaceae; g__Ruminococcus | 0.23 (0.06) | 0.02 (0.01) |
|  | p__Firmicutes; c__Clostridia; o__Clostridiales; f__Lachnospiraceae;  g__Dorea | 0.50 (0.04) | 0.32 (0.03) |
|  | p__Firmicutes; c__Clostridia; o__Clostridiales; f__Peptostreptococcaceae; g__ | 0.51 (0.06) | 0.68 (0.05) |
|  | p__Bacteroidetes; c__Bacteroidia; o__Bacteroidales; f__Porphyromonadaceae; g__Parabacteroides | 0.55 (0.07) | 0.38 (0.05) |
|  | p__Firmicutes; c__Clostridia; o__Clostridiales; f__Ruminococcaceae; g__Faecalibacterium | 0.06 (0.01) | 0.13 (0.02) |
|  | p__Firmicutes; c__Clostridia; o__Clostridiales; f__Ruminococcaceae; g__Ruminococcus | 0.19 (0.03) | 0.09 (0.02) |
|  | p__Firmicutes; c__Clostridia; o__Clostridiales; f__Lachnospiraceae; g__[Ruminococcus] | 0.17 (0.02) | 0.17 (0.05) |
|  | p__Firmicutes; c__Clostridia; o__Clostridiales; f__Lachnospiraceae | 0.03 (0.01) | 0.08 (0.01) |
|  | p__Firmicutes; c__Clostridia; o__Clostridiales; f__Lachnospiraceae;  g__Roseburia | 2.76 (0.20) | 2.17 (0.16) |
|  | p__Firmicutes; c__Clostridia; o__Clostridiales; f__Ruminococcaceae;  g__ | 0.17 (0.02) | 0.09 (0.01) |
|  | p__Proteobacteria; c__Deltaproteobacteria; o__Desulfovibrionales; f__Desulfovibrionaceae; g__Desulfovibrio | 0.44 (0.09) | 0.17 (0.03) |
|  | p__Firmicutes; c__Clostridia; o__Clostridiales; f__Ruminococcaceae;  g__Oscillospira | 0.71 (0.09) | 0.86 (0.07) |
|  | p__Firmicutes; c__Clostridia; o__Clostridiales; f__Peptostreptococcaceae; g__ | 1.82 (0.17) | 2.61 (0.20) |
|  | p__Bacteroidetes; c__Bacteroidia; o__Bacteroidales; f__Prevotellaceae;  g__Prevotella | 0.99 (0.15) | 1.08 (0.14) |
| **All_Male** | p__Firmicutes; c__Clostridia; o__Clostridiales; f__Clostridiaceae;  g__ | 0.27 (0.12) | 0.84 (0.12) |
|  | p__Firmicutes; c__Bacilli; o__Lactobacillales; f__Streptococcaceae; g__Streptococcus | 1.17 (0.27) | 0.52 (0.10) |
|  | p__Firmicutes; c__Clostridia; o__Clostridiales; f__Lachnospiraceae | 0.79 (0.16) | 0.27 (0.05) |
|  | p__Firmicutes; c__Clostridia; o__Clostridiales; f__Lachnospiraceae;  g__Dorea | 0.96 (0.14) | 0.43 (0.05) |
|  | p__Firmicutes; c__Clostridia; o__Clostridiales; f__Ruminococcaceae;  g__Oscillospira | 0.27 (0.12) | 0.84 (0.12) |
|  | p__Firmicutes; c__Clostridia; o__Clostridiales; f__Ruminococcaceae; g__Ruminococcus | 0.23 (0.06) | 0.10 (0.02) |
|  | p__Firmicutes; c__Clostridia; o__Clostridiales; f__Lachnospiraceae;  g__Coprococcus | 1.38 (0.20) | 0.59 (0.05) |
|  | p__Firmicutes; c__Clostridia; o__Clostridiales; f__Lachnospiraceae;  g__ | 1.91 (0.44) | 0.81 (0.10) |
|  | p__Proteobacteria; c__Gammaproteobacteria; o__Enterobacteriales; f__Enterobacteriaceae | 1.06 (0.88) | 3.07 (0.51) |
|  | p__Firmicutes; c__Clostridia; o__Clostridiales; f__Ruminococcaceae | 0.27 (0.12) | 0.84 (0.12) |
|  | p__Firmicutes; c__Clostridia; o__Clostridiales; f__Lachnospiraceae;  g__Coprococcus | 1.38 (0.24) | 0.62 (0.06) |
|  | p__Firmicutes; c__Clostridia; o__Clostridiales; f__Lachnospiraceae;  g__ | 0.13 (0.03) | 0.05 (0.01) |
|  | p__Bacteroidetes; c__Bacteroidia; o__Bacteroidales; f__Prevotellaceae;  g__Prevotella | 0.55 (0.24) | 1.17 (0.22) |
|  | p__Firmicutes; c__Clostridia; o__Clostridiales; f__Lachnospiraceae;  g__Blautia | 2.41 (0.35) | 1.15 (0.11) |
| **All_Female** | p__Proteobacteria; c__Gammaproteobacteria; o__Enterobacteriales; f__Enterobacteriaceae | 1.02 (0.22) | 2.15 (0.33) |
|  | p__Firmicutes; c__Clostridia; o__Clostridiales; f__Clostridiaceae；  g__SMB53 | 0.20 (0.03) | 0.38 (0.05) |
|  | p__Firmicutes; c__Clostridia; o__Clostridiales; f__Ruminococcaceae；  g__Oscillospira | 0.83 (0.11) | 1.22 (0.12) |
|  | p__Firmicutes; c__Clostridia; o__Clostridiales; f__Clostridiaceae | 0.60 (0.09) | 1.43 (0.21) |
|  | p__Firmicutes; c__Clostridia; o__Clostridiales; f__Lachnospiraceae；  g__Dorea | 0.40 (0.04) | 0.19 (0.02) |
|  | p__Firmicutes; c__Clostridia; o__Clostridiales; f__Veillonellaceae；  g__Megasphaera | 0.58 (0.11) | 0.32 (0.09) |
|  | p__Firmicutes; c__Clostridia; o__Clostridiales; f__Lachnospiraceae；  g__Roseburia | 2.54 (0.20) | 2.05 (0.26) |
|  | p__Firmicutes; c__Bacilli; o__Turicibacterales; f__Turicibacteraceae；  g__Turicibacter | 0.17 (0.04) | 0.29 (0.04) |
|  | p__Firmicutes; c__Bacilli; o__Lactobacillales; f__Leuconostocaceae；  g__ | 0.06 (0.03) | 0.29 (0.12) |
|  | p__Firmicutes; c__Clostridia; o__Clostridiales; f__Clostridiaceae；  g__ | 0.93 (0.23) | 1.43 (0.20) |
|  | p__Firmicutes; c__Clostridia; o__Clostridiales; f__Peptostreptococcaceae；g__ | 0.44 (0.06) | 0.66 (0.07) |
|  | p__Firmicutes; c__Clostridia; o__Clostridiales; f__Ruminococcaceae；g__Faecalibacterium | 0.05 (0.01) | 0.16 (0.03) |
|  | p__Bacteroidetes; c__Bacteroidia; o__Bacteroidales; f__Porphyromonadaceae；g__Parabacteroides | 0.58 (0.08) | 0.39 (0.08) |
|  | p__Firmicutes; c__Clostridia; o__Clostridiales; f__Ruminococcaceae | 0.16 (0.03) | 0.53 (0.18) |
| **RSA** | p__Firmicutes; c__Erysipelotrichi; o__Erysipelotrichales; f__Erysipelotrichaceae; g__ | 0.17 (0.05) | 0.64 (0.12) |
|  | p__Firmicutes; c__Clostridia; o__Clostridiales; f__Veillonellaceae; g__Phascolarctobacterium | 0.16 (0.06) | 0.43 (0.09) |
|  | p__Firmicutes; c__Clostridia; o__Clostridiales; f__Veillonellaceae;  g__Dialister | 3.01 (0.40) | 1.28 (0.25) |
|  | p__Firmicutes; c__Clostridia; o__Clostridiales; f__Lachnospiraceae;  g__Coprococcus | 0.15 (0.02) | 0.43 (0.07) |
|  | p__Firmicutes; c__Clostridia; o__Clostridiales; f__Lachnospiraceae; g__[Ruminococcus] | 0.05 (0.02) | 0.41 (0.20) |
| **Ghana** | p__Firmicutes; c__Clostridia; o__Clostridiales; f__Clostridiaceae;  g__SMB53 | 0.22 (0.06) | 0.54 (0.06) |
|  | p__Firmicutes; c__Bacilli; o__Turicibacterales; f__Turicibacteraceae;  g__Turicibacter | 0.20 (0.07) | 0.40 (0.05) |
|  | p__Firmicutes; c__Clostridia; o__Clostridiales; f__Ruminococcaceae; g__Faecalibacterium | 0.32 (0.09) | 0.12 (0.02) |
|  | p__Firmicutes; c__Clostridia; o__Clostridiales; f__Lachnospiraceae;  g__Blautia | 1.12 (0.23) | 1.20 (0.24) |
|  | p__Firmicutes; c__Bacilli; o__Lactobacillales; f__Streptococcaceae;  g__Streptococcus | 0.78 (0.33) | 0.19 (0.04) |
| **Group glucose** | | **Elevated fasting plasma glucose** | **Non- elevated fasting plasma glucose** |
| **All** | p__Proteobacteria; c__Gammaproteobacteria; o__Enterobacteriales; f__Enterobacteriaceae | 2.91 (0.43) | 1.38 (0.20) |
|  | p__Firmicutes; c__Bacilli; o__Lactobacillales; f__Leuconostocaceae;  g__ | 0.21 (0.05) | 0.17 (0.06) |
|  | p__Firmicutes; c__Bacilli; o__Lactobacillales; f__Streptococcaceae;  g__Streptococcus | 0.31 (0.06) | 0.25 (0.09) |
|  | p__Firmicutes; c__Clostridia; o__Clostridiales; f__Veillonellaceae;  g__Dialister | 0.48 (0.10) | 0.97 (0.11) |
|  | p__Firmicutes; c__Erysipelotrichi; o__Erysipelotrichales; f__Erysipelotrichaceae; g__Bulleidia | 0.13 (0.02) | 0.21 (0.02) |
| **All_Male** | p__Actinobacteria; c__Actinobacteria; o__Bifidobacteriales; f__Bifidobacteriaceae; g__Bifidobacterium | 0.46 (0.15) | 0.91 (0.20) |
|  | p__Firmicutes; c__Bacilli; o__Lactobacillales; f__Streptococcaceae;  g__Streptococcus | 0.49 (0.13) | 0.44 (0.23) |
|  | p__Firmicutes; c__Clostridia; o__Clostridiales; f__Ruminococcaceae; g__Faecalibacterium | 0.34 (0.08) | 0.53 (0.08) |
|  | p__Firmicutes; c__Bacilli; o__Lactobacillales; f__Leuconostocaceae;  g__ | 0.29 (0.08) | 0.17 (0.07) |
| **All_Female** | p__Proteobacteria; c__Gammaproteobacteria; o__Enterobacteriales; f__Enterobacteriaceae | 1.98 (0.33) | 1.26 (0.23) |
|  | p__Firmicutes; c__Erysipelotrichi; o__Erysipelotrichales; f__Erysipelotrichaceae | 0.92 (0.23) | 1.74 (0.22) |
|  | p__Firmicutes; c__Clostridia; o__Clostridiales; f__Veillonellaceae | 0.50 (0.15) | 1.16 (0.16) |
|  | p__Firmicutes; c__Erysipelotrichi; o__Erysipelotrichales; f__Erysipelotrichaceae | 0.09 (0.02) | 0.20 (0.02) |
|  | p__Proteobacteria; c__Deltaproteobacteria; o__Desulfovibrionales; f__Desulfovibrionaceae | 0.26 (0.11) | 0.37 (0.07) |
|  | p__Firmicutes; c__Clostridia; o__Clostridiales; f__[Mogibacteriaceae] | 0.05 (0.01) | 0.14 (0.02) |
|  | p__Firmicutes; c__Bacilli; o__Lactobacillales; f__Leuconostocaceae | 0.15 (0.05) | 0.17 (0.08) |
| **RSA** | p__Bacteroidetes; c__Bacteroidia; o__Bacteroidales; f__Bacteroidaceae; g__Bacteroides | 0.74 (0.42) | 0.08 (0.03) |
| **Jamaica** | p__Firmicutes; c__Clostridia; o__Clostridiales; f__Ruminococcaceae;  g__ | 2.04 (0.51) | 0.98 (0.33) |
| **Group blood pressure** | | **Elevated blood pressure** | **Non- Elevated blood pressure** |
| **All** | p__Firmicutes; c__Clostridia; o__Clostridiales; f__Ruminococcaceae;  g__Oscillospira | 0.48 (0.11) | 0.88 (0.07) |
|  | p__Proteobacteria; c__Gammaproteobacteria; o__Enterobacteriales; f__Enterobacteriaceae | 1.82 (0.58) | 2.01 (0.22) |
|  | p__Firmicutes; c__Clostridia; o__Clostridiales; f__Lachnospiraceae; g__[Ruminococcus] | 0.35 (0.07) | 0.29 (0.06) |
|  | p__Firmicutes; c__Clostridia; o__Clostridiales; f__Clostridiaceae; g__ | 0.58 (0.12) | 0.96 (0.09) |
|  | p__Firmicutes; c__Clostridia; o__Clostridiales; f__Lachnospiraceae | 0.53 (0.11) | 0.21 (0.03) |
|  | p__Bacteroidetes; c__Bacteroidia; o__Bacteroidales; f__Porphyromonadaceae; g__Parabacteroides | 0.65 (0.11) | 0.39 (0.04) |
|  | p__Firmicutes; c__Clostridia; o__Clostridiales; f__Lachnospiraceae; g__Dorea | 0.66 (0.07) | 0.32 (0.02) |
|  | p__Firmicutes; c__Clostridia; o__Clostridiales; f__Ruminococcaceae; g__Ruminococcus | 0.06 (0.03) | 0.46 (0.11) |
|  | p__Firmicutes; c__Clostridia; o__Clostridiales; f__Lachnospiraceae; g__Lachnospira | 0.04 (0.02) | 0.14 (0.02) |
|  | p__Firmicutes; c__Clostridia; o__Clostridiales; f__; g__ | 0.12 (0.04) | 0.36 (0.06) |
|  | p__Firmicutes; c__Clostridia; o__Clostridiales; f__Ruminococcaceae; g__ | 0.05 (0.01) | 0.15 (0.01) |
|  | p__Firmicutes; c__Clostridia; o__Clostridiales; f__Lachnospiraceae; g__ | 0.07 (0.01) | 0.02(0.003) |
|  | p__Firmicutes; c__Clostridia; o__Clostridiales; f__Ruminococcaceae; g__Ruminococcus | 0.18 (0.04) | 0.12 (0.02) |
|  | p__Firmicutes; c__Clostridia; o__Clostridiales; f__Ruminococcaceae; g__ | 0.14 (0.02) | 0.12 (0.01) |
| **All_Male** | p__Firmicutes; c__Clostridia; o__Clostridiales; f__Lachnospiraceae; g__[Ruminococcus] | 0.48 (0.12) | 0.36 (0.12) |
|  | p__Firmicutes; c__Clostridia; o__Clostridiales; f__Ruminococcaceae; g__Faecalibacterium | 1.22 (0.23) | 2.13 (0.19) |
|  | p__Firmicutes; c__Bacilli; o__Lactobacillales; f__Streptococcaceae; g__Streptococcus | 0.06 (0.04) | 0.62 (0.19) |
| **All_Female** | p__Bacteroidetes; c__Bacteroidia; o__Bacteroidales; f__Porphyromonadaceae; g__Parabacteroides | 0.56 (0.26) | 1.70 (0.22) |
|  | p__Proteobacteria; c__Gammaproteobacteria; o__Enterobacteriales; f__Enterobacteriaceae | 1.37 (0.29) | 2.25 (0.18) |
|  | p__Firmicutes; c__Clostridia; o__Clostridiales; f__Peptostreptococcaceae; g__ | 0.56 (0.15) | 1.09 (0.09) |
|  | p__Firmicutes; c__Clostridia; o__Clostridiales; f__Ruminococcaceae; g__Oscillospira | 0.47 (0.16) | 1.28 (0.19) |
|  | p__Firmicutes; c__Clostridia; o__Clostridiales; f__Clostridiaceae; g__ | 2.73 (0.61) | 1.40 (0.15) |
|  | p__Bacteroidetes; c__Bacteroidia; o__Bacteroidales; f__Bacteroidaceae; g__Bacteroides | 0.60 (0.19) | 1.03 (0.12) |
|  | p__Firmicutes; c__Clostridia; o__Clostridiales; f__Clostridiaceae; g__ | 0.14 (0.07) | 0.24 (0.03) |
|  | p__Firmicutes; c__Bacilli; o__Turicibacterales; f__Turicibacteraceae; g__Turicibacter | 0.57 (0.20) | 0.16 (0.02) |
|  | p__Firmicutes; c__Clostridia; o__Clostridiales; f__Lachnospiraceae | 0.20 (0.05) | 0.12 (0.02) |
|  | p__Firmicutes; c__Clostridia; o__Clostridiales; f__Ruminococcaceae; g__Ruminococcus |  |  |
| **Group HDL** | | **Low HDL** | **High HDL** |
| **All** | p__Firmicutes; c__Clostridia; o__Clostridiales; f__Ruminococcaceae; g__Ruminococcus | 0.74 (0.22) | 0.14 (0.03) |
|  | p__Firmicutes; c__Clostridia; o__Clostridiales; f__Ruminococcaceae; g__Faecalibacterium | 0.11 (0.01) | 0.09 (0.02) |
|  | p__Proteobacteria; c__Gammaproteobacteria; o__Enterobacteriales; f__Enterobacteriaceae | 2.20 (0.35) | 2.02 (0.32) |
| **All_Male** | p__Firmicutes; c__Clostridia; o__Clostridiales; f__Ruminococcaceae; g__Ruminococcus | 0.76 (0.30) | 0.11 (0.04) |
| **RSA** | p__Firmicutes; c__Clostridia; o__Clostridiales; f__Veillonellaceae; g__Megasphaera | 1.25 (0.27) | 0.53 (0.13) |
| **Group triglyceride** | | **Hyper-triglyceridemia** | **Non-hyper-triglyceridemia** |
| **All** | p__Firmicutes; c__Clostridia; o__Clostridiales; f__Ruminococcaceae; g__Oscillospira | 0.26 (0.07) | 0.89 (0.07) |
|  | p__Bacteroidetes; c__Bacteroidia; o__Bacteroidales; f__Prevotellaceae; g__Prevotella | 0.82 (0.23) | 1.95 (0.17) |
|  | p__Firmicutes; c__Clostridia; o__Clostridiales; f__Ruminococcaceae | 0.26 (0.14) | 0.08 (0.01) |
|  | p__Firmicutes; c__Clostridia; o__Clostridiales; f__Ruminococcaceae; g__Oscillospira | 0.06 (0.03) | 0.20 (0.02) |
|  | p__Firmicutes; c__Clostridia; o__Clostridiales; f__Lachnospiraceae; g__[Ruminococcus] | 0.33 (0.09) | 0.29 (0.06) |
|  | p__Firmicutes; c__Clostridia; o__Clostridiales; f__Veillonellaceae; g__Megasphaera | 0.92 (0.40) | 0.24 (0.06) |
|  | p__Bacteroidetes; c__Bacteroidia; o__Bacteroidales; f__Bacteroidaceae; g__Bacteroides | 0.60 (0.25) | 0.27 (0.07) |
|  | p__Firmicutes; c__Clostridia; o__Clostridiales; f__Ruminococcaceae; g__Ruminococcus | 0.27 (0.07) | 0.13 (0.02) |
|  | p__Firmicutes; c__Clostridia; o__Clostridiales; f__Ruminococcaceae | 0.08 (0.02) | 0.03 (0.01) |
|  | p__Firmicutes;c__Clostridia;o__Clostridiales;f__Ruminococcaceae;g__ | 0.05 (0.01) | 0.17 (0.01) |
|  | p__Bacteroidetes;c__Bacteroidia;o__Bacteroidales;f__Porphyromonadaceae;g__Parabacteroides | 0.75 (0.18) | 0.39 (0.04) |
| **All_Female** | p__Firmicutes; c__Clostridia; o__Clostridiales; f__Ruminococcaceae; g__Oscillospira | 0.20 (0.10) | 1.16 (0.10) |
|  | p__Bacteroidetes; c__Bacteroidia; o__Bacteroidales; f__Bacteroidaceae; g__Bacteroides | 1.02 (0.45) | 0.31 (0.11) |
|  | p__Bacteroidetes; c__Bacteroidia; o__Bacteroidales; f__Porphyromonadaceae; g__Parabacteroides | 1.10 (0.32) | 0.41 (0.06) |
|  | p__Firmicutes; c__Clostridia; o__Clostridiales; f__Ruminococcaceae; g__Ruminococcus | 0.41 (0.19) | 0.16 (0.05) |
|  | p__Firmicutes; c__Clostridia; o__Clostridiales; f__Lachnospiraceae; g__[Ruminococcus] | 0.49 (0.16) | 0.19 (0.07) |
|  | p__Firmicutes; c__Clostridia; o__Clostridiales; f__Lachnospiraceae; g__Coprococcus | 0.39 (0.11) | 0.12 (0.03) |
|  | p__Firmicutes; c__Clostridia; o__Clostridiales; f__Ruminococcaceae | 0.44 (0.27) | 0.09 (0.02) |
|  | p__Bacteroidetes; c__Bacteroidia; o__Bacteroidales; f__Prevotellaceae; g__Prevotella | 0.77 (0.34) | 1.97 (0.22) |
|  | p__Firmicutes; c__Clostridia; o__Clostridiales; f__Ruminococcaceae; g__Oscillospira | 0.06 (0.05) | 0.26 (0.03) |
|  | p__Firmicutes; c__Clostridia; o__Clostridiales; f__Lachnospiraceae; g__Coprococcus | 1.26 (0.30) | 0.44 (0.06) |
|  | p__Firmicutes; c__Clostridia; o__Clostridiales; f__Ruminococcaceae; g__Ruminococcus | 0.40 (0.11) | 0.12 (0.02) |
|  | p__Bacteroidetes; c__Bacteroidia; o__Bacteroidales; f__Bacteroidaceae; g__Bacteroides | 0.47 (0.16) | 0.17 (0.04) |
|  | p__Bacteroidetes; c__Bacteroidia; o__Bacteroidales; f__Bacteroidaceae; g__Bacteroides | 3.26 (0.91) | 1.41 (0.18) |
|  | p__Firmicutes; c__Clostridia; o__Clostridiales; f__Clostridiaceae;  g__ | 0.21 (0.13) | 1.06 (0.14) |
|  | p__Firmicutes; c__Erysipelotrichi; o__Erysipelotrichales;  f__Erysipelotrichaceae; g__Catenibacterium | 1.30 (0.79) | 1.56 (0.19) |
|  | p__Firmicutes; c__Clostridia; o__Clostridiales; f__Ruminococcaceae;  g__ | 0.04 (0.02) | 0.23 (0.02) |
|  | p__Firmicutes; c__Clostridia; o__Clostridiales; f__Ruminococcaceae; g__Ruminococcus | 0.11 (0.03) | 0.04 (0.01) |
|  | p__Firmicutes; c__Clostridia; o__Clostridiales; f__Ruminococcaceae;  g__Oscillospira | 0.15 (0.05) | 0.05 (0.01) |
|  | p__Firmicutes; c__Clostridia; o__Clostridiales; f__Lachnospiraceae | 0.80 (0.31) | 0.18 (0.03) |
|  | p__Bacteroidetes; c__Bacteroidia; o__Bacteroidales; f__Rikenellaceae;  g__ | 0.45 (0.15) | 0.21 (0.04) |
|  | p__Firmicutes; c__Clostridia; o__Clostridiales; f__Ruminococcaceae;  g__Oscillospira | 0.09 (0.03) | 0.04 (0.01) |
|  | p__Firmicutes; c__Clostridia; o__Clostridiales; f__Clostridiaceae;  g__SMB53 | 0.09 (0.05) | 0.30 (0.03) |
|  | p__Bacteroidetes; c__Bacteroidia; o__Bacteroidales; f__Bacteroidaceae; g__Bacteroides | 0.62 (0.38) | 0.23 (0.05) |
|  | p__Firmicutes; c__Clostridia; o__Clostridiales; f__Ruminococcaceae;  g__ | 0.11 (0.09) | 0.43 (0.05) |
|  | p__Proteobacteria; c__Betaproteobacteria; o__Burkholderiales;  f__Alcaligenaceae; g__Sutterella | 0.21 (0.10) | 0.05 (0.02) |
|  | p__Firmicutes; c__Clostridia; o__Clostridiales; f__Lachnospiraceae; g__[Ruminococcus] | 0.22 (0.07) | 0.10 (0.02) |
|  | p__Firmicutes; c__Clostridia; o__Clostridiales; f__Clostridiaceae;  g__Clostridium | 0.41 (0.14) | 0.13 (0.02) |
| **USA** | p__Firmicutes; c__Clostridia;o__Clostridiales;f__Veillonellaceae;  g__Acidaminococcus | 0.79 (0.30) | 0.23 (0.06) |
| **Ghana** | p__Firmicutes; c__Clostridia; o__Clostridiales; f__Lachnospiraceae;  g__Coprococcus | 0.33 (0.13) | 0.09 (0.02) |
| **RSA** | p__Firmicutes; c__Clostridia; o__Clostridiales; f__Lachnospiraceae; g__[Ruminococcus] | 0.34 (0.10) | 0.24 (0.13) |
|  | p__Firmicutes; c__Clostridia; o__Clostridiales; f__Ruminococcaceae;  g__Oscillospira | 0.33 (0.14) | 1.24 (0.13) |

*Number in red, ESVs were significantly enriched in patients with one of the CM risk factors.
