# Supplementary material for: The human microbiota is associated with cardiometabolic risk across the epidemiologic transition
Source: PLoS One. 2019 Jul 24;14(7):e0215262. doi: 10.1371/journal.pone.0215262 (PMC6656343; doi:10.1371/journal.pone.0215262)
Supplement: S3 Table — USA, the United States of America; RSA, South Africa. (DOCX) [file pone.0215262.s025.docx]

**S3 Table.** **Contributions of CM risk factors and participants’ characteristics to the interindividual dissimilarities in oral microbiota composition.** USA, the United States of America; RSA, South Africa.

| **Supplementary Table 3a. Oral microbiota (weighted UniFrac distance)** | | | | | | | | |
| --- | --- | --- | --- | --- | --- | --- | --- | --- |
|  | **USA** | | **RSA** | | **Ghana** | | **Jamaica** | |
|  | **R^2^** | **P** | **R^2^** | **P** | **R^2^** | **P** | **R^2^** | **P** |
| **Waist circumference** | 0.018 | 0.007 | 0.009 | 0.204 | 0.010 | 0.073 | 0.018 | 0.180 |
| **Elevated blood Pressure** | 0.003 | 0.727 | 0.006 | 0.423 | 0.011 | 0.047 | 0.007 | 0.654 |
| **Elevated fasting plasma glucose** | 0.004 | 0.543 | 0.003 | 0.811 | 0.006 | 0.280 | / | / |
| **HDL concentration** | 0.005 | 0.413 | 0.011 | 0.113 | 0.005 | 0.448 | / | / |
| **Elevated fasting plasma glucose** | 0.013 | 0.036 | 0.009 | 0.187 | 0.008 | 0.163 | 0.018 | 0.195 |
| **Gender** | 0.025 | 0.001 | 0.007 | 0.364 | 0.028 | 0.001 | 0.011 | 0.456 |
| **Age** | 0.020 | 0.036 | 0.005 | 0.964 | 0.009 | 0.603 | 0.060 | 0.011 |
| **BMI** | 0.010 | 0.496 | 0.011 | 0.518 | 0.012 | 0.316 | 0.069 | 0.006 |
| **Sleep** | 0.007 | 0.281 | 0.013 | 0.040 | 0.015 | 0.016 | 0.009 | 0.591 |
| **Smoke** | 0.071 | 0.001 | 0.017 | 0.519 | 0.006 | 0.885 | 0.073 | 0.080 |
| **Alcohol consumption** | 0.021 | 0.002 | 0.006 | 0.420 | 0.011 | 0.049 | 0.006 | 0.739 |
|  |  |  |  |  |  |  |  |  |
| **Supplementary Table 3b. Oral microbiota (unweighted UniFrac distance)** | | | | | | | | |
|  | **USA** | | **RSA** | | **Ghana** | | **Jamaica** | |
|  | **R^2^** | **P** | **R^2^** | **P** | **R^2^** | **P** | **R^2^** | **P** |
| **Waist circumference** | 0.016 | 0.009 | 0.015 | 0.015 | 0.013 | 0.022 | 0.016 | 0.244 |
| **Elevated blood Pressure** | 0.005 | 0.557 | 0.002 | 0.974 | 0.004 | 0.541 | 0.009 | 0.608 |
| **Elevated fasting plasma glucose** | 0.006 | 0.393 | 0.001 | 0.960 | 0.004 | 0.634 | / | / |
| **HDL concentration** | 0.011 | 0.040 | 0.005 | 0.537 | 0.007 | 0.195 | / | / |
| **Elevated fasting plasma glucose** | 0.010 | 0.067 | 0.006 | 0.467 | 0.004 | 0.628 | 0.010 | 0.526 |
| **Gender** | 0.030 | 0.001 | 0.016 | 0.014 | 0.009 | 0.101 | 0.029 | 0.017 |
| **Age** | 0.019 | 0.031 | 0.016 | 0.208 | 0.011 | 0.402 | 0.046 | 0.024 |
| **BMI** | 0.014 | 0.213 | 0.018 | 0.108 | 0.013 | 0.270 | 0.029 | 0.021 |
| **Sleep** | 0.005 | 0.484 | 0.007 | 0.349 | 0.005 | 0.544 | 0.046 | 0.023 |
| **Smoke** | 0.077 | 0.001 | 0.020 | 0.263 | 0.007 | 0.633 | 0.055 | 0.007 |
| **Alcohol consumption** | 0.016 | 0.008 | 0.018 | 0.006 | 0.006 | 0.292 | 0.016 | 0.217 |
